# Establishment of subcutaneous transplantation platform for delivering induced pluripotent stem cell-derived insulin-producing cells

**DOI:** 10.1371/journal.pone.0318204

**Published:** 2025-01-30

**Authors:** Hong Thuan Tran, Watchareewan Rodprasert, Irma Padeta, Saranyou Oontawee, Steven dwi Purbantoro, Anatcha Thongsit, Parkpoom Siriarchavatana, Sayamon Srisuwatanasagul, Hiroshi Egusa, Thanaphum Osathanon, Chenphop Sawangmake

**Affiliations:** 1 Second Century Fund (C2F) Chulalongkorn University for Doctoral Scholarship, Chulalongkorn University, Bangkok, Thailand; 2 Faculty of Veterinary Science, The International Graduate Program of Veterinary Science and Technology (VST), Chulalongkorn University, Bangkok, Thailand; 3 Faculty of Veterinary Science, Veterinary Clinical Stem Cell and Bioengineering Research Unit, Chulalongkorn University, Bangkok, Thailand; 4 Faculty of Veterinary Science, Veterinary Stem Cell and Bioengineering Innovation Center (VSCBIC), Chulalongkorn University, Bangkok, Thailand; 5 Faculty of Veterinary Medicine, Western University, Kanchanaburi, Thailand; 6 Faculty of Veterinary Science, Department of Anatomy, Chulalongkorn University, Bangkok, Thailand; 7 Division of Molecular and Regenerative Prosthodontics, Center for Advanced Stem Cell and Regenerative Research, Tohoku University Graduate School of Dentistry, Sendai, Japan; 8 Faculty of Dentistry, Dental Stem Cell Biology Research Unit and Department of Anatomy, Chulalongkorn University, Bangkok, Thailand; 9 Faculty of Dentistry, Center of Excellence in Regenerative Dentistry, Chulalongkorn University, Bangkok, Thailand; 10 Faculty of Veterinary Science, Department of Pharmacology, Chulalongkorn University, Bangkok, Thailand; Osaka University, JAPAN

## Abstract

Potential trend of regenerative treatment for type I diabetes has been introduced for more than a decade. However, the technologies regarding insulin-producing cell (IPC) production and transplantation are still being developed. Here, we propose the potential IPC production protocol employing mouse gingival fibroblast-derived induced pluripotent stem cells (mGF-iPSCs) as a resource and the pre-clinical approved subcutaneous IPC transplantation platform for further clinical confirmation study. With a multi-step induction protocol, the functional and matured IPCs were generated by 13 days with a long-term survival capability. Further double encapsulation of mGF-iPSC-derived IPCs (mGF-iPSC-IPCs) could preserve the insulin secretion capacity and the transplantation potential of the generated IPCs. To address the potential on IPC transplantation, a 2-step subcutaneous transplantation procedure was established, comprising 1) vascularized subcutaneous pocket formation and 2) encapsulated IPC bead transplantation. The *in vivo* testing confirmed the safety and efficiency of the platform along with less inflammatory response which may help minimize tissue reaction and graft rejection. Further preliminary *in vivo* testing on subcutaneous IPC-bead transplantation in an induced type I diabetic mouse model showed beneficial trends on blood glucose control and survival rate sustainability of diabetic mice. Taken together, an established mGF-iPSC-IPC generation protocol in this study will be the potential backbone for developing the iPSC-derived IPC production employing human and animal cell resources. As well as the potential further development of IPC transplantation platform for diabetes treatment in human and veterinary practices using an established subcutaneous encapsulated IPC-bead transplantation platform presented in this study.

## Introduction

Regenerative treatment for type I diabetes has been introduced for more than a decade. It started from the cadaveric islet transplantation in type I diabetic patients according to an Edmonton protocol [1–3]. The successful clinical protocol could control hyperglycemic condition without an exogenous insulin administration for a year [4]. Nevertheless, this protocol requires cadaveric islet donors and immunosuppressive agent administration lifelong [4]. To address these burdens, stem cell-based regenerative treatment for type I diabetes has been introduced. In this regard, various insulin-producing cells (IPCs) production platforms have been established and validated aiming for safe and efficient IPC generation [5–11]. However, the induction efficiency, cost-effectiveness, and IPC production yield are the main factors affecting the achievement of clinically applicable IPCs [12–14].

To achieve the potential IPCs for further clinical application, the induced pluripotent stem cells (iPSCs) have been proposed as a potential cell resource for generative the efficient IPCs in terms of induction efficiency and production yield [10,15,16]. Hence, the iPSCs possess high capacities of cell proliferation and differentiation potential, iPSCs from several sources have been employed for generating the IPCs [10,17,18]. In this study, the iPSCs generated from mouse gingival fibroblasts (mGFs) of C57BL/6J mice (mGF-iPSCs) were used as a cell resource for generating the transplantable IPCs [19]. It should be noted that the trend of autologous or patients’ own cell therapy has been proposed to minimize the risk of tissue rejection after transplanted [20]. This leads to numerous studies working on iPSC generation from various tissues of human and animal origins [10,17,19,21–24]. In this regard, oral tissue-derived cells are considered as the promising resources for derivation of mesenchymal stem cells (MSCs) and stromal cells used for iPSC generation [5,6,19,25–27]. Successful iPSC generation from donors’ tissues is a key success for further development of patient-specific cell therapy and allogeneic cell transplant in HLA-matched recipients [28].

In terms of IPC production, the clinical applicable IPCs should be a mature population of pancreatic islet lineages with high capacities of glucose-dependent insulin secretion and long-term survival rate [5–8,12,29]. To further sustain the transplantation potential of the IPCs, micro- and macro-encapsulation techniques have been proposed [12,30–32]. Our previous reports suggested that an optimized IPC encapsulation provided an optimal diffusion of glucose and insulin through the encapsulation membrane which was crucial to a glucose-dependent insulin secretion from the encapsulated IPCs [12,30]. Furthermore, the double encapsulation technique may help minimize the local and systemic inflammatory responses to the transplanted IPCs and prevent the potential tissue reactions and graft rejection [8,12,30,33,34]. Along with additional techniques on transplantation site preparation, an increased success rate of IPC transplantation has been reported [35].

Thus, the generation of mGF-iPSC-derived IPCs (mGF-iPSC-IPCs) was fully established and validated in this study along with the production of ready-to-transplant IPCs using double encapsulation technique. To study the potential application of the generated IPCs for diabetes treatment, a preliminary transplantation study was conducted in an induced type I diabetic mouse model with an established 2-step subcutaneous transplantation platform. The safety and efficiency of the IPC generation and transplantation platforms established in this study will be the potential backbones for further development of stem cell-based regenerative therapy for diabetes in both human and veterinary practices.

## Materials and methods

### mGF-iPSC culture and embryoid body generation

The mGF-iPSCs were established and reported in the previous study [36]. These mGF-iPSC colonies were a kind gift from Prof. Hiroshi Egusa (Tohoku University, Japan). These cells were successfully reprogrammed using the transfection of *Oct3/4*, *Sox2*, and *Klf4*, and underwent the full characterization as mentioned in the report.

To cultivate the mGF-iPSCs, the feeder cells were seeded in a medium consisting of high-glucose Dulbecco’s Modified Eagle Medium (DMEM) (Thermo Fisher Scientific, USA) supplemented with 1% of Antibiotic-Antimycotic (Thermo Fisher Scientific), 1% GlutaMax (Thermo Fisher Scientific), and 7% fetal bovine serum (FBS) (Thermo Fisher Scientific). The feeder cells were inactivated by 6 μg/mL Mitomycin C (Nacalai Tesque, Japan). Next, the mGF-iPSCs were cultured on the inactivated feeder cell layer continuously. They were maintained in growth medium containing high-glucose DMEM supplemented with 1% Antibiotics-Antimycotic, 1% GlutaMax, and 15% FBS (Gibco, US standard, USA), 2% β-mercaptoethanol (Sigma-Aldrich, USA), and 1% non-essential amino acids (NEAAs) (Sigma-Aldrich). All cells were incubated at 37°C, 5% CO_2_, and humidified condition. The medium was changed every 48 hours.

For embryoid body generation, the mGF-iPSC colonies were trypsinized and seeded onto low-attachment culture containers to form the embryoid bodies (EBs). The EB colonies were maintained in growth medium and with medium changing every 48 hours for further experiments.

### Pancreatic IPC production

The mGF-iPSC-derived IPC (mGF-iPSC-IPC) production protocol was modified regarding a previous study [37]. With a core 6-step induction protocol along with major and minor variations established in this study, mGF-iPSC-IPCs were successfully generated and characterized. The 6-step induction protocols were developed with 3 major variations named P-iPS 1.1, P-iPS 1.2, and P-iPS 1.3. These 3 major variations employed different types of containers for EB formation and IPC differentiation including non-treated cell culture dish (Eppendorf, USA) (P-iPS 1.1), Petri Dish (MicroQC, Thailand) (P-iPS 1.2), and Petri Dish (Corning, France) (P-iPS 1.3).

After EB formation, all EB colonies were then differentiated toward pancreatic lineages using further 5 induction steps. Briefly, the protocols started from definitive endoderm (DE) induction with DMEM supplemented with 1% GlutaMax, 0.1% bovine serum albumin (BSA) (Sigma-Aldrich), and 50 ng/mL activin A (Sigma-Aldrich) for 3 days. Then, cells were continually cultured with pancreatic progenitor (PP) induction medium for 2 days with DMEM supplemented with 0.5% BSA (Sigma-Aldrich), 1% insulin-transferrin-selenium (ITS) (Invitrogen, USA), and 2 mM retinoic acid (Sigma-Aldrich). The third step was pancreatic endocrine (PE) induction with DMEM-low glucose (Invitrogen) supplemented with 0.5% BSA, 1% ITS, 10 ng/mL basic fibroblast growth factor (bFGF) (R&D, USA), and 20 ng/mL epidermal growth factor (EGF) (Millipore corporation) for 3 days. The fourth and fifth steps were insulin-producing cell (IPC) induction with DMEM/F12 (Invitrogen) supplemented with 1% ITS, 10 ng/mL bFGF, and 10 nM nicotinamide (Sigma-Aldrich) for 3 days, then followed by the IPC maturation step for additional 2 days.

In this study, the protocol titled P-iPS 1.3 was further variated into 2 major variations (P-iPS 1.3.1 and P-iPS 1.3.2). For protocol P-iPS 1.3.1, PP induction step was added with 1 mM taurine (Sigma-Aldrich) and 10 nM nicotinamide. PE induction step was added with 1 μM *γ*-secretase inhibitor (DAPT) (Sigma-Aldrich). IPC induction step was added with 100 nM glucagon-like peptide (GLP)-1 (Sigma-Aldrich) and 1 μM DAPT. IPC maturation step was added with 100 nM GLP-1, 1 μM DAPT, and 30 μM dopamine hydrochloride (DA) (Sigma-Aldrich). Additionally, 2% β-mercaptoethanol (Sigma-Aldrich) and 1% non-essential amino acids (NEAAs) (Sigma-Aldrich) were supplemented in induction media starting from DE induction till IPC maturation.

The protocol P-iPS 1.3.2 was formulated as the above details with the withdrawal of DA during the IPC maturation.

### Functional testing for IPCs

During IPC induction, the cells/colonies were tested for functional property by the glucose-stimulated C-peptide secretion (GSCS) assay. Cells/colonies were maintained in Krebs-Ringer Bicarbonate HEPES (KRBH) buffer comprising the mixture of 120 mM NaCl, 5 mM KCl, 2.5 mM CaCl_2_, 1.1 mM MgCl_2_, 25 mM NaHCO_3_, and 10 mM HEPES, then adjust to pH 7.4 by NaOH solution [5].

Cells/colonies were then challenged with a series of glucose-containing KRBH buffer starting from 0-, 5.5-, and 22-mM glucose, respectively. The duration of each stimulation was 60 minutes. The anhydrous glucose (Sigma-Aldrich) was used to prepare the challenging solutions. Functional properties of the IPCs were illustrated as the relative value of C-peptide secretion (ng)/DNA amount of IPCs (mg)/stimulation time (60 minutes).

### Reverse transcription-quantitative polymerase chain reaction (RT-qPCR)

The total RNA of iPSC-derived IPCs were collected and extracted by using TRIzol-RNA isolation reagent (Thermo Fisher Scientific) and DirectZol-RNA extraction kit (Zymo Research, USA), respectively, following the optimized protocol. Subsequently, the total RNA was converted into complementary DNA (cDNA) with ImProm-TM Reverse Transcription System (Promega, USA). The RT-qPCR assay was run by employing PowerUp™ SYBR™ Green Master Mix (Thermo Fisher Scientific) in a real-time PCR Detection System (Bio-Rad, USA) for pancreatic IPC-related targeted genes. The primer sets in this study are shown in S1 Table. The ribosomal Protein L13 (*Rlp13A*) was employed as a reference gene to normalize the targeted relative mRNA expression by calculating the 2^-ΔΔCt^, whereas ΔΔCt = (Ct^target gene^ − Ct^Rlp13A^)treated − (Ct^target gene^ − Ct^Rlp13A^)control. The Ct value is referred to cycle threshold.

### Double layers of IPC encapsulation

The double encapsulation of IPCs was performed by resuspending day 13 of iPSC-derived IPCs in 2% (w/v) alginate solution (Sigma-Aldrich) and dripped into a 30% (w/v) of Pluronic-F127 (Sigma-Aldrich) as beads with the following optimized protocol [30]. The beads were maintained in the IPC induction medium at 37°C with 5% CO_2_. The carrier beads without IPCs were employed as a control for further experiments.

### Alginate/Pluronic-F127 bead dissolution

Dissolution in this study was performed by incubating the beads in dissolving buffer consisting of 0.2-M C_6_H_5_Na_2_O_7_·2H_2_O, pH 7.4 and 0.1-M EDTA for 5 minutes at 37°C. The degraded beads’ layers were washed with PBS buffer for 3 times with 3 minutes each time.

### Immunocytochemistry staining

The IPCs were fixed with 100% cold methanol (−20°C) for 15 minutes at 4°C, and permeabilized for 1 minute using 0.1% Triton^®^X-100 (VWR life science, USA) in PBS at room temperature. The fixed and permeabilized IPCs were blocked with 10% donkey serum in PBS for 1 hour at 4°C. The monoclonal mouse clone Pro-Insulin C-Pep-01 (Millipore) and anti-mouse Insulin monoclonal (Merck, USA) were employed with dilution at 1:100 in 1% BSA and incubated with the IPCs for 24 hours at 4°C. After 24 hours, the IPCs were washed with PBS and incubated with anti-mouse goat secondary IgG conjugated to fluorescein isothiocyanate isomer 1 (Bio-rad, USA) with dilution at 1:1000 in 1% BSA in the dark environment at room temperature for 1 hour. The nucleus was stained with DAPI (0.4 μg/mL) (Sigma-Aldrich). The labeled-IPCs were detected under 100x and 200x magnifications of a fluorescent microscope incorporated with Carl Zeiss^™^ Apotome.2 apparatus (Carl Zeiss, Germany).

### Live/Dead-cell staining

The collected iPSC-derived IPCs were evaluated for its viability by double staining with Calcein AM chemical (Invitrogen) and Ethidium homodimer (Invitrogen) with dilution at 1:1000 and incubated for 30 minutes. The stained samples were washed with PBS and observed under the 100x magnification of a fluorescent microscope incorporated with Carl Zeiss^™^ Apotome.2 apparatus (Carl Zeiss, Germany).

### Animal husbandry

All the protocols and experimental animals in this study were strictly following the recommendations in the Guide for Care and Use of Laboratory Animals of National Institutes of Health. The protocol was approved by the Institutional Animal Care and Use Committee, Chulalongkorn University Laboratory Animal Center (CULAC), Faculty of Veterinary Science, Chulalongkorn University (Protocol Number: 2173018). All male *C57BL/6NJcl* mice were purchased from Nomura Siam International Co., Ltd, Thailand. The mice were housed in a ventilated cage system under the standard room condition and fed with standard pellet mouse diet and *ad libitum* water. Each mouse was closely monitored.

The euthanasia was performed by using CO_2_ (30–70%) inhalation and cervical dislocation according to the American Veterinary Medical Association (AVMA) Guidelines for the Euthanasia of Animals: 2020 Edition (CITE). The blood and organs were then collected for further experiments.

### Subcutaneous transplantation site preparation

In this experiment, the mice were divided into two groups: i) CTRL group and ii) Carrier-bead group. In the Carrier-bead group, retaining space was made by injecting a sterile mixture of 250 ng/mL Vascular Endothelial Growth Factor-165 (VEGF-165) (GenScript, USA) and 10% Pluronic-F127 (Sigma-Aldrich) with and implanting an 18-G catheter (Nipro, Thailand) subcutaneously on the back of the mice, aseptically, under general anesthesia induced with isoflurane inhalation. After 14 days of the subcutaneous pocket formation, approximately twenty to twenty-five carrier-beads without cells were assigned to be transplanted. The mice were observed for 21 days (short-term study) and 42 days (long-term study). For CTRL group, the mice were treated for catheter implantation only. All mice were checked for their health and weight every week. In addition, all mice were given 6 hours of water and feed deprivation prior to blood collection.

### Diabetic induction and IPC-bead transplantation

In this experiment, two groups of mice: i) Sham group and ii) IPC-bead Tx group were employed. The mice with normal fasting blood glucose (FBG) (<11.1 mM) were assigned to be diabetic through intraperitoneal administration of streptozotocin (STZ) (Sigma-Aldrich) at 180 mg/kg in Citrate buffer, pH 4.5 (Sigma-Aldrich). Only mice with non-FBG (N-FBG) level exceeded 20 mM considered as diabetic after seven days induction and further assigned for transplantation. Then, the subcutaneous transplantation site formation was performed as mentioned above. At the time of transplantation, 20–25 beads with IPCs were equivalently transplanted into the subcutaneous transplantation site in the IPC-bead Tx group under general anesthesia. On another hand, the Sham group was transplanted with carried beads without cells. After transplantation, all mice were checked for their health and weight every week. In addition, the mice were deprived for water and feed for 6 hours prior to blood collection for FBG, HOMA-IR/beta analysis, and intraperitoneal glucose tolerance test (IPGTT). At termination day, all mice were humanely euthanized as mentioned above and intracardiac blood was collected. In addition, the pancreas, transplantation sites (skin and all subcutaneous tissue at the insertion area), kidney, and brain were harvested for histological analysis.

### HOMA index analysis

The HOMA index consisting of HOMA-beta, HOMA-IR and QUICKI are calculated based on the levels of C-peptide in the plasma. The C-peptide levels were detected using the Rat/Mouse C-peptide enzyme-linked immunosorbent assay kit (Millipore, USA) according to the manufacturer’s protocol. The value was calculated following the formula: HOMA-beta for normal mice = fasting C − peptide × 20 ÷ (FBG − 3.5) or HOMA-beta for diabetic mice = fasting C − peptide × 20 ÷ (FBG − 3.5) + 50 and HOMA − IR = fasting C − peptide × FBG ÷ 22.5; and $QUICKI=\frac{1}{log(HOMA-IR)}$. The unit of C-peptide and FBG are μIU/mL and (mmoL/mL), respectively.

### Intraperitoneal injection glucose tolerance test (IPGTT)

The mice were fasted for 4–6 hours to maintain a low blood glucose status. After fasting, an initial blood draw was done before each glucose challenge. The glucose challenge was performed by injecting the mice with glucose (1.5 g/kg) intraperitoneally. FBG were evaluated at 15, 30, 60, and 120 minutes after glucose challenge. Blood glucose was measured by a glucose meter (Accu-Chek, Roche, Switzerland).

### Histopathology

Collected tissues were rinsed with 1x phosphate buffered saline (PBS), pH 7.4 and fixed in 4% Paraformaldehyde (PFA) for 24 hours. Then, the tissues were dehydrated in a graded ethanol series, embedded in paraffin wax, and sectioned at a 30 μm thickness. Three serial sections of each sample were stained with hematoxylin and eosin (H&E), anti-rabbit Insulin monoclonal (Cell Signaling Technology, USA), and anti-mouse CD31/PECAM-1 monoclonal (Santa Cruz Biotechnology, USA) for histopathological examination. Stained slides were digitally visualized with a light microscope (Olympus Group, Japan). A blind evaluation was performed independently to the experimental groups.

### Cytokine analysis

The profile of proteins in plasma samples was detected with an antibody array of mouse inflammation channel (ab133999, abcam, USA) following the manufacturer’s protocol. Briefly, antibody array membranes were blocked in blocking buffer for 30 minutes prior to incubation of 1 mL of the samples overnight at 4°C. The sample solution was then discarded from each container, and the membranes were washed with wash buffer. Another washing with wash buffer were performed with shaking for another 3 times at room temperature. The membranes were then incubated in 1:1000 diluted streptavidin–horseradish peroxidase at room temperature for 2 hours, washed thoroughly and exposed to a peroxidase substrate prior to imaging. A chemiluminescence image was taken with an Amersham imager 600 (UV), Japan, with a 2-minute exposure. As a result, the list of proteins’ detections in the membrane array was matched with the provided cytokine map. A semi-quantitative analysis was performed by measuring the intensity of dot clots with ImageJ software (National Institutes of Health, NIH). A heatmap was generated using R software (Bell Lab, GNU project). The intensity was calculated by the formula $X=(X_{\left(y\right)}-negative)\times \frac{P1}{P_{\left(y\right)}}$ following the manufacturer’s protocol.

### Complete blood count (CBC) and blood chemistry (BC) analysis

Blood was withdrawn and collected into the EDTA or heparin (BD Vacutainer^®^, Thailand) blood collecting-tubes. The plasma was collected after centrifuging at 2,500 rpm for 15 minutes at 4°C. The parameters of blood chemistry (BC) were checked in the panel of comprehensive cards by a biochemistry analyzer, the Vetscan VS2 machine (Abaxis, Zoetis, UK). The CBC was detected in the CBC panel by RIA company, Bangkok, Thailand.

### Data analysis

All statistical analysis were done using SPSS statistics 22 software (IBM Corporation, USA). Mann-Whitney U test was used for comparing two independent samples. Differences were considered statistically significant if the *p*-value was <0.05. Graphs were all made with GraphPad Prism 9.0 (GraphPad Software, Inc., San Diego, CA).

## Results

### Establishment and optimization of the effective IPC differentiation using mGF-iPSC modeling

To set up the potential iPSC-based type I DM treatment model, mGF-iPSCs were used in this study. The mGF-iPSCs were established and reported in the previous study [36]. These mGF-iPSC colonies were a kind gift from Prof. Hiroshi Egusa (Tohoku University, Japan). These cells were successfully reprogrammed using the transfection of *Oct3/4*, *Sox2*, and *Klf4*, and underwent the full characterization as mentioned in the report.

After colony expansion, the representative mGF-iPSCs were validated for their morphology, stemness marker expression, and alkaline phosphatase activity (S1 Fig).

**Fig 1:**
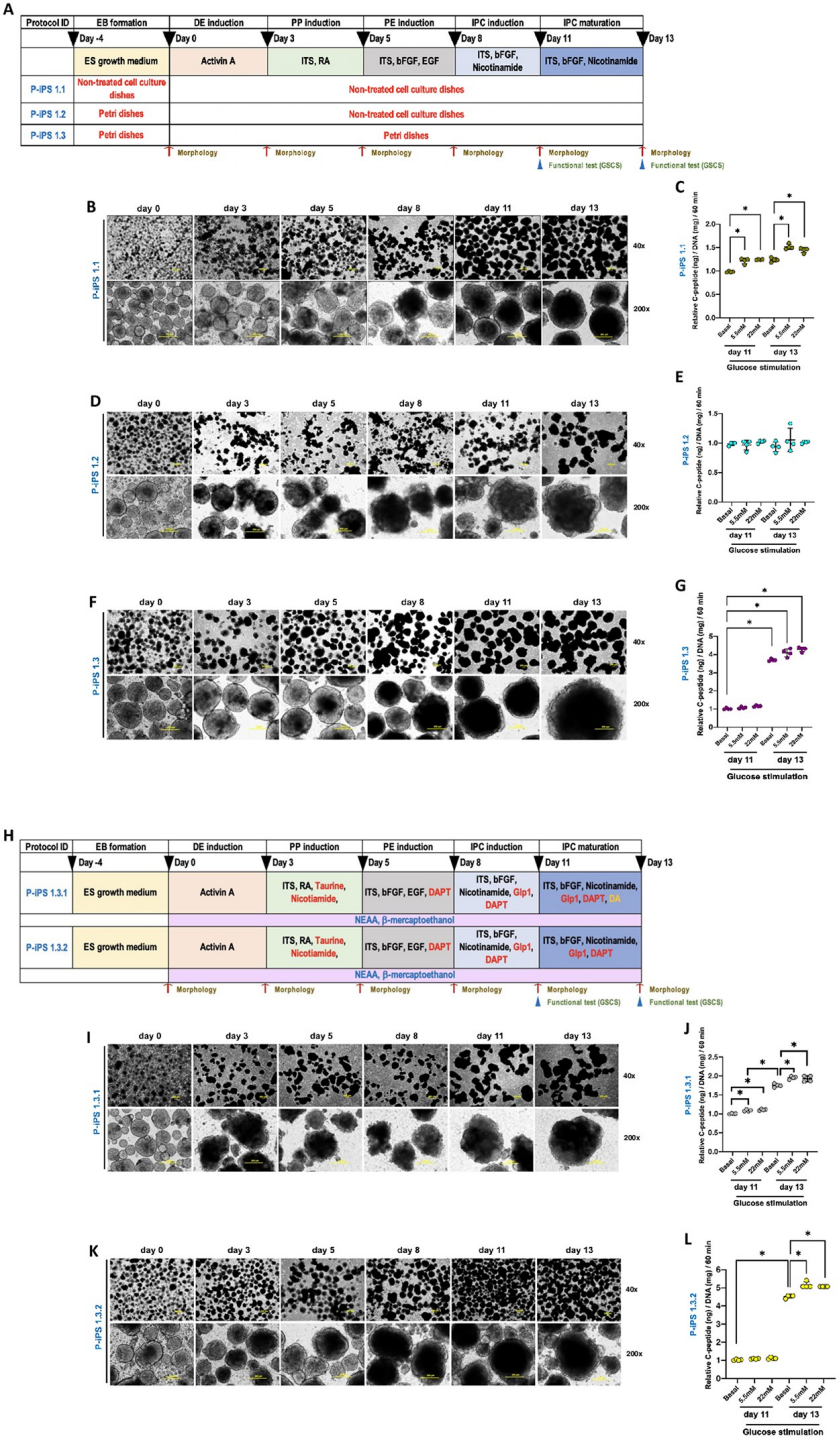
Establishment and optimization of the effective IPC differentiation using mGF-iPSC modeling. **(A)** Schematic presentation of the 6-step mGF-iPSC-IPC generation protocols with 3 major variations named P-iPS 1.1, P-iPS 1.2, and P-iPS 1.3. **(B)**, **(D)**, **(F)** Morphological appearances of the mGF-iPSC-IPC colonies generated from protocol P-iPS 1.1, P-iPS 1.2, and P-iPS 1.3 at magnification of 40x and 200x. **(C)**, **(E)**, **(G)** Glucose-stimulated C-peptide secretion (GSCS) analysis of mGF-iPSC-IPCs generated from protocol P-iPS 1.1, P-iPS 1.2, and P-iPS 1.3. **(H)** Schematic presentation of the 6-step mGF-iPSC-IPC generation protocols variated from protocol P-iPS 1.3 named P-iPS 1.3.1 and P-iPS 1.3.2. **(I)**, **(K)** Morphological appearances of the mGF-iPSC-IPC colonies generated from protocol P-iPS 1.3.1 and P-iPS 1.3.2 at magnification of 40x and 200x. **(J)**, **(L)** GSCS analysis of mGF-iPSC-IPCs generated from protocol P-iPS 1.3.1 and P-iPS 1.3.2. ***Abbreviations*:** *embryoid bodies (EB)*, *definitive endoderm (DE)*, *pancreatic progenitor (PP)*, *pancreatic endocrine (PE)*, *insulin-producing cell (IPC)*.

The 6-step iPSC derived-IPC differentiation protocols were developed with 3 major variations named P-iPS 1.1, P-iPS 1.2, and P-iPS 1.3 (Fig 1A). These 3 major variations employed different types of containers for EB formation and IPC differentiation. To assess the efficiency of IPC production, colony morphology, and relative C-peptide secretion using GSCS assay was performed with basal (0 mM), 5.5 mM, and 22 mM glucose stimulation, respectively. The results showed that all protocols could generate IPC colonies upon the induction process. However, the protocol P-iPS 1.1 and P-iPS 1.2 were insufficient to deliver the efficient relative C-peptide secretion upon glucose stimulation when compared to protocol P-iPS 1.3 (Fig 1B–1E). The latter protocol gave a dose-dependent pattern of relative C-peptide secretion upon glucose stimulation, and the basal C-peptide level of day-11 IPC was higher than day-13 IPC around 3.5 folds, preliminarily suggesting the maturation of IPC population (Fig 1F and 1G).

To further improve the protocol efficiency, protocol P-iPS 1.3 was then modified and designated into 2 variations: P-iPS 1.3.1 and P-iPS 1.3.2 (Fig 1H). The results showed that protocol P-iPS 1.3.2 exerted higher efficiency on IPC production compared with another variation in term of relative C-peptide secretion. The basal relative C-peptide secretion of IPCs harvested from protocol P-iPS 1.3.2 at day 13 was elevated more than 4 folds comparing with day-11 IPCs (Fig 1I–1L). Together with the trend of higher colony amount as noticed in the colony pictures, the protocol P-iPS 1.3.2 was selected for IPC production in this study and subsequently analyzed for IPC characteristics.

### An established *in vitro* induction protocol yields efficient and long-term IPC survival

**Fig 2:**
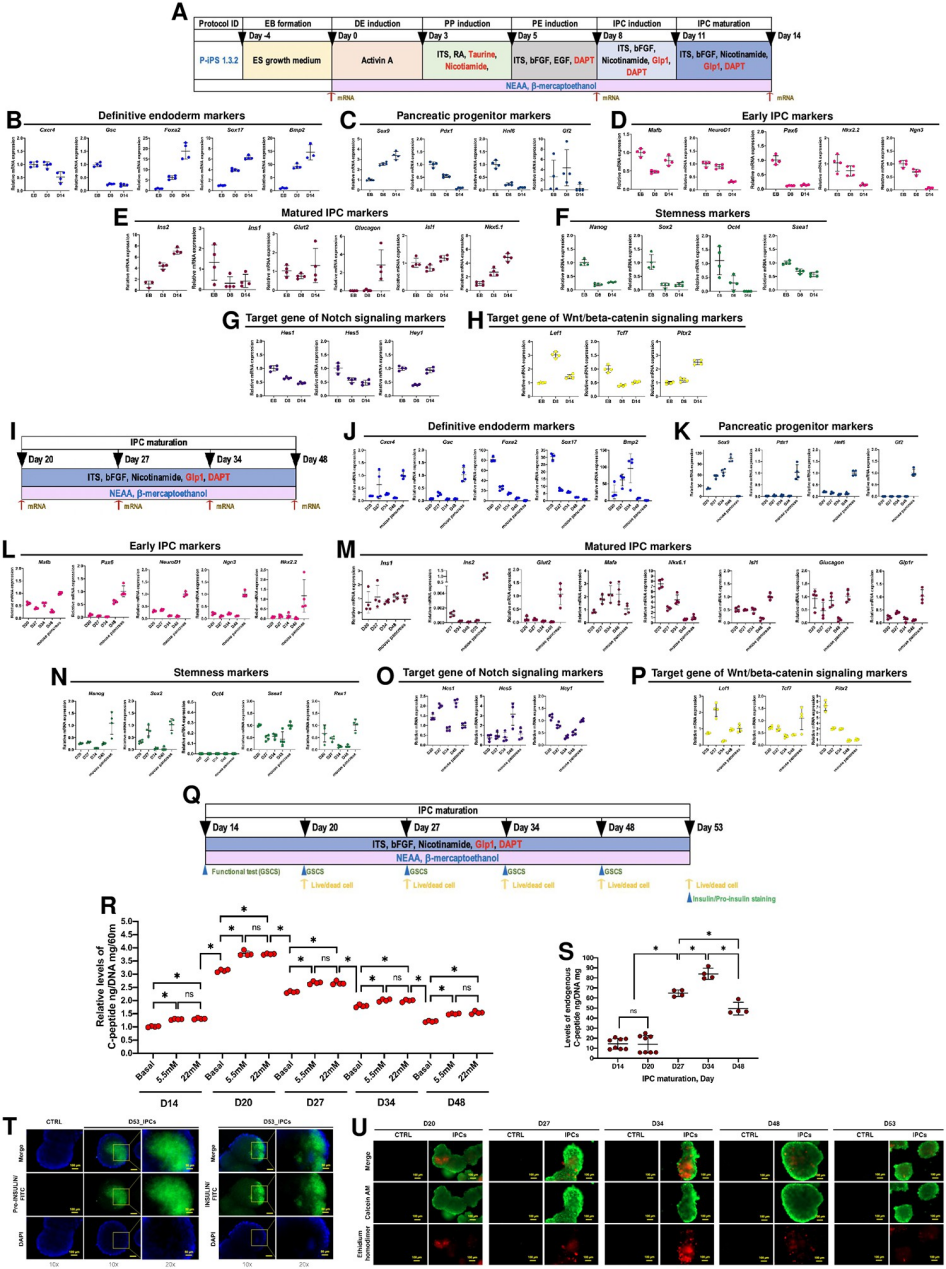
Genotypic characteristics of the potential mGF-iPSC-derived IPCs (mGF-iPSC-IPCs) and long-term maintenance of the matured IPCs. **(A)** Schematic presentation of a potential 6-step mGF-iPSC-IPC generation protocol named P-iPS 1.3.2 and mRNA analysis timepoints. **(B)**, **(C)**, **(D)**, **(E)**, **(F)**, **(G)**, **(H)** Relative mRNA expression levels of pancreatic markers (definitive endoderm, pancreatic progenitor, early IPC, and matured IPC), stemness markers, and related signaling pathways (Notch and Wnt/beta-catenin). **(I)** Schematic presentation of long-term maintenance of the matured IPCs and mRNA analysis timepoints. **(J)**, **(K)**, **(L)**, **(M)**, **(N)**, **(O)**, **(P)** Relative mRNA expression levels of pancreatic markers (definitive endoderm, pancreatic progenitor, early IPC, and matured IPC), stemness markers, and related signaling pathways (Notch and Wnt/beta-catenin) upon long-term maintenance of the matured IPCs. **(Q)** Schematic presentation of long-term maintenance of the matured IPCs and timepoints for glucose-stimulated C-peptide secretion (GSCS), live/dead, and pro-insulin and insulin staining analyses. **(R)** GSCS analysis of matured IPCs generated from protocol P-iPS 1.3.2 upon long-term maintenance. **(S)** Endogenous C-peptide levels of matured IPCs generated from protocol P-iPS 1.3.2 upon long-term maintenance. **(T)** Pro-insulin and insulin immunocytochemistry (ICC) staining of matured IPCs generated from protocol P-iPS 1.3.2 upon long-term maintenance at magnification of 100x and 200x. **(U)** Live/dead staining of matured IPCs generated from protocol P-iPS 1.3.2 upon long-term maintenance.

To fully clarify the genotypic characteristics of the mGF-iPSC-derived IPCs (mGF-iPSC-IPCs) generated from protocol P-iPS 1.3.2, mRNA expressions regarding pancreatic markers (definitive endoderm, pancreatic progenitor, early IPC, and matured IPC), stemness markers, and related signaling pathways (Notch and Wnt/beta-catenin) were analyzed using RT-qPCR at the pivotal differentiation stages (embryoid body formation: D0, pancreatic endoderm induction: D8, and IPC maturation: D14) (Fig 2A). The primer sets were designed and validated (S1 Table). The results illustrated the upregulation of core matured IPC markers upon the induction process (*Ins2*, *Glut2*, *Glucagon*, and *Nkx6*.*1*), while the sets of definitive endoderm, pancreatic progenitor, and early IPC markers were mostly downregulated upon the induction (Fig 2B–2E). In this regard, stemness markers were downregulated suggesting the maturation process of the IPC generation (Fig 2F). Besides, fluctuations of Notch and Wnt/beta-catenin target genes were observed which revealed the influence of these two signaling upon the differentiation process (Fig 2G and 2H). The results support the efficiency of the established IPC induction protocol.

To obtain the transplantable IPCs, the sustainability of the generated IPCs is crucial. In this regard, long-term maintenance of the matured IPCs was conducted. Sets of mRNA markers previously mentioned were periodically analyzed till day 48 (Fig 2I). Isolated mouse pancreatic mRNA (*C57BL/6NJcl* strain) was used as a control. The results showed that the IPCs could sustain the expression of core early IPC markers (*Mafb*, *Pax6*, *NeuroD1*, *Ngn3*, and *Nkx2*.*2*) and matured IPC markers (*Ins1*, *Ins2*, *Glut2*, *Mafa*, and *Isl1*) during the maintenance period, while the expression of stemness mRNA markers was still low suggesting the genotypic maintenance of the matured IPCs (Fig 2J–2N). Additionally, the expression of Notch and Wnt/beta-catenin target genes were comparable to the mouse pancreas control suggesting the relevance of these signaling with the IPC maintenance (Fig 2O and 2P).

Further interval analyses on functional properties and viability of the maintained IPCs were conducted using GSCS, endogenous C-peptide, and Live/Dead assays. Additional staining of pro-insulin and insulin were performed when the maintenance period ended (Fig 2Q). The results illustrated that the IPCs could maintain the glucose concentration-dependent response on C-peptide secretion throughout the maintenance period (Fig 2R). It should be noted that basal C-peptide secretion on day 20 was increased and then eventually declined to the normal level. However, the endogenous C-peptide levels of the IPCs after day 20 were continually increased which suggested the capacity of insulin production (Fig 2S).

To further confirm the presence of pro-insulin and insulin, ICC was used to specifically stain the IPCs. The results revealed that the IPCs could maintain the expression of pro-insulin and insulin until the maintenance period ended (Fig 2T). Additional Live/Dead assay showed the viability of the majority of the IPC population, while minor dead population was found (Fig 2U). These results support the long-term survival and efficient functioning of the IPCs generated *in vitro*.

### IPC encapsulation preserves insulin secretion capacity and transplantation potential

**Fig 3:**
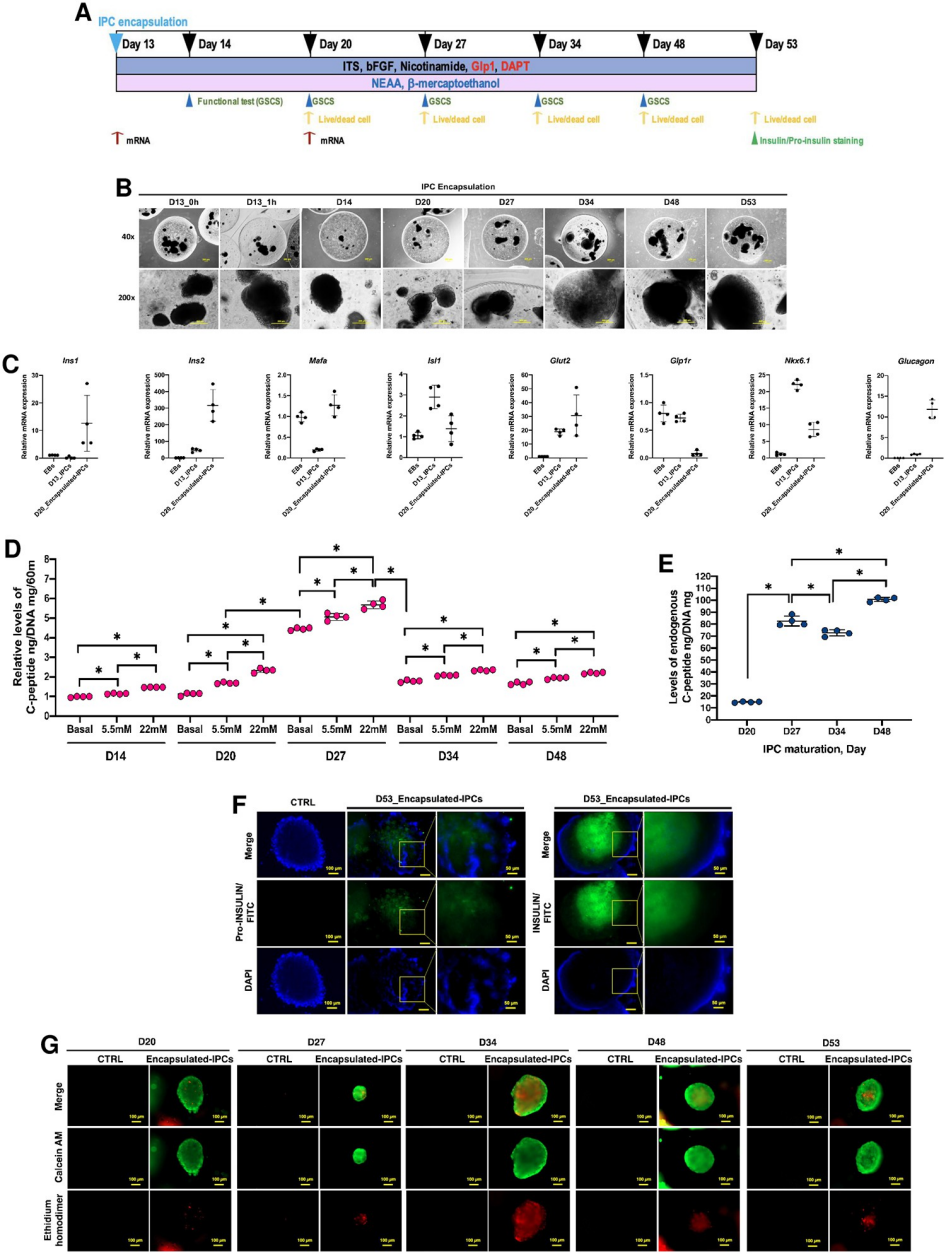
IPC encapsulation preserves insulin secretion capacity and transplantation potential. **(A)** Schematic presentation of the matured IPC encapsulation and timepoints for glucose-stimulated C-peptide secretion (GSCS), live/dead, and pro-insulin and insulin staining analyses. **(B)** Morphological appearances of the encapsulated IPCs upon long-term maintenance at magnification of 40x and 200x. **(C)** Relative mRNA expression levels of important pancreatic markers of the encapsulated IPCs upon long-term maintenance. **(D)** GSCS analysis of the encapsulated IPCs upon long-term maintenance. **(E)** Endogenous C-peptide levels of the encapsulated IPCs upon long-term maintenance. **(F)** Pro-insulin and insulin immunocytochemistry (ICC) staining of the encapsulated IPCs upon long-term maintenance at magnification of 100x and 200x. **(G)** Live/dead staining of the encapsulated IPCs upon long-term maintenance.

To minimize the immune response in host model and sustain the insulin secretion capacity, the double encapsulation of IPCs using biocompatible materials (alginate and Pluronic-F127) was applied according to our previous report [30]. IPC morphology, genotypic characteristic, functional properties, and viability were periodically assessed throughout the maintenance period (Fig 3A).

After the encapsulation, the IPCs remained intact and stayed inside the encapsulated beads throughout the maintenance period (Fig 3B). Representative genotypic characteristics focusing on matured IPC mRNA markers were analyzed by benchmarking the encapsulated IPCs (day 20) with the unencapsulated IPCs (day 13) and the EBs (day 0). The results showed that the IPCs could maintain the expression of key matured IPC markers under the encapsulation condition (Fig 3C). Further analyses on functional properties were conducted using GSCS and endogenous C-peptide assays. It was interesting that the encapsulated IPCs could maintain the glucose concentration-dependent C-peptide secretion along the maintenance period. However, the surge of basal C-peptide level on day 27 was found before returning to normal level (Fig 3D). Further analysis showed the continually increasing of endogenous C-peptide, confirming the insulin production capacity of the encapsulated IPCs (Fig 3E).

The presence of pro-insulin and insulin was confirmed using ICC assay as illustrated in Fig 3F, suggesting the capability of insulin production throughout the maintenance period. Additional Live/Dead assay suggested that the majority of encapsulated IPC population was viable, while minor dead population was found (Fig 3G). These results demonstrate that the encapsulation preserves the insulin secretion capacity and the transplantation potential of the IPCs.

### Establishment of subcutaneous transplantation platform for efficient IPC transplantation

**Fig 4:**
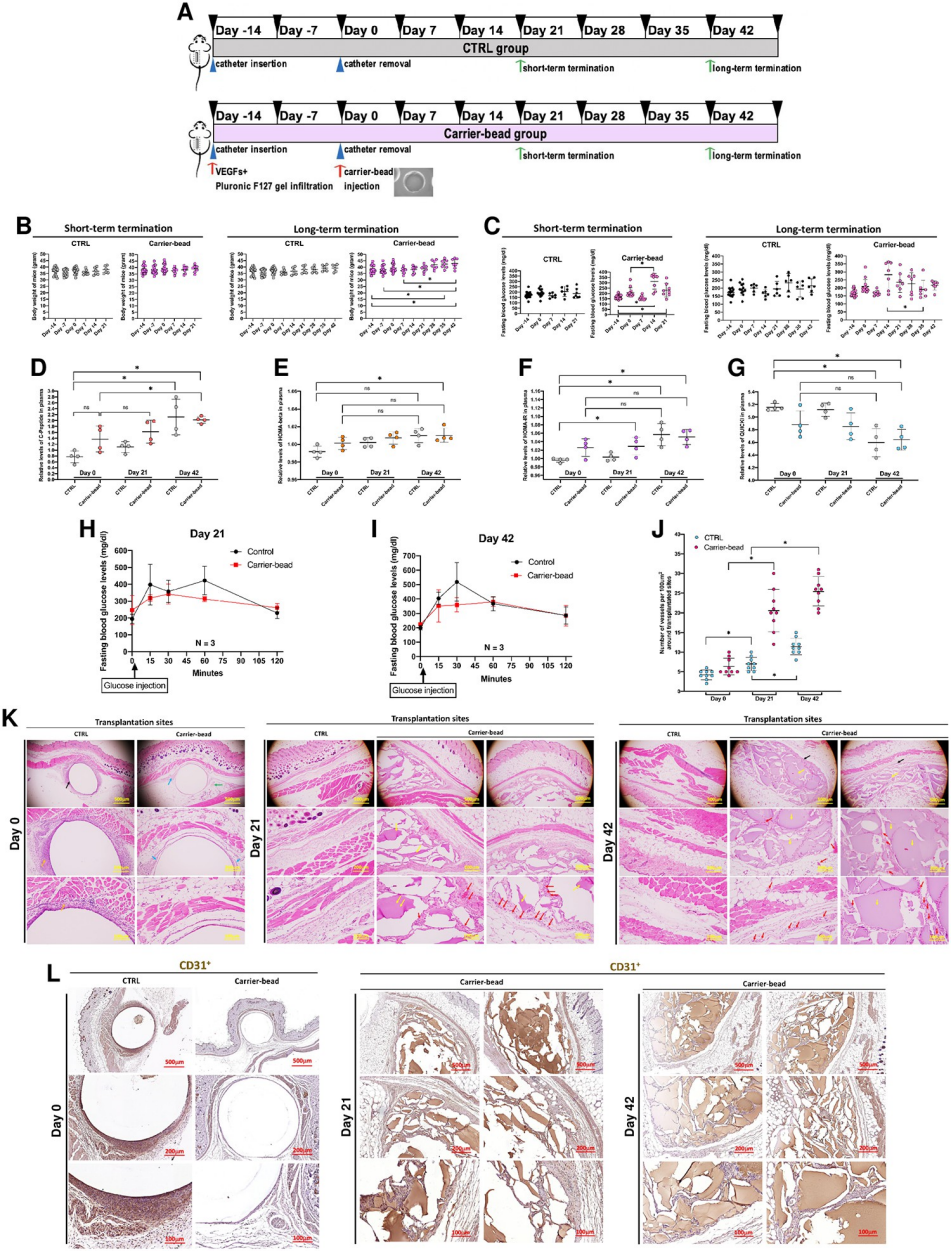
Establishment of subcutaneous transplantation platform for efficient IPC transplantation. **(A)** Schematic presentation of the establishment of subcutaneous IPC transplantation platform. **(B)** Body weight of animals during the study period. **(C)** Fasting blood glucose (FBG) levels of animals during the study period. **(D)**, **(E)**, **(F)**, **(G)** Relative C-peptide levels, HOMA-beta, HOMA-IR, and QUICKI indices of animals during the study. **(H)**, **(I)** Intraperitoneal glucose tolerance test (IPGTT) of animals at day 21 and 42 of the study. **(J)** Blood vessel counting of animals during the study. **(K)** Histopathological examination of subcutaneous transplantation site at day 0, 21, and 42 of the study using hematoxylin & eosin (H&E) staining. **(L)** Immunohistochemistry (IHC) analysis for CD31 of transplantation site at day 0, 21, and 42 of the study. ***Annotations*:** *black arrows*: *inserted catheter sheath*, *orange arrows*: *thick connective tissues*, *blue arrows*: *thin connective tissues*, *green arrows*: *adipose tissues*, *yellow arrows transplanted beads*, *red arrows*: *blood vessels*.

Subcutaneous transplantation platform for delivering encapsulated IPCs was established using a 2-step transplantation protocol comprising 1) vascularized subcutaneous pocket formation and 2) encapsulated IPC bead transplantation. The vascularized subcutaneous pocket formation was conducted by the placement of an 18-G catheter tube (1 cm in length) underneath the skin along with the subcutaneous infusion of pocket retaining gel (sterile mixture of 250 ng/mL VEGF-165 + 10% Pluronic-F127 gel) at the same area. Fourteen days later, the catheter tube was removed, and the encapsulated beads were transplanted into the pocket area (S2 Fig). In this phase, 36 *C57BL/6NJcl* mice were divided into 2 groups: i) the control (CTRL) group (n = 18) (only catheter tube placement) and ii) the carrier-bead group (n = 18) (catheter tube placement + pocket retaining gel infusion + blank bead transplant). Short-term (21 days) and long-term (42 days) observations were conducted to validate the transplantation platform formation efficiency (Fig 4A).

Weekly body weight and fasting blood glucose level were observed throughout the study period. The body weight gain of animals in CTRL and carrier-bead groups were normal in both short-term and long-term studies (Fig 4B), while the fasting blood glucose levels were slightly increased in carrier-bead group around day 14 of the study before declining to the normal level (Fig 4C). Additionally, the complete blood count (CBC), and blood chemistry (BC) parameters of both groups were not significantly different throughout the study period (S2 and S3 Tables).

To observe the effects of transplantation platform on insulin sensitivity and glucose control, series of insulin sensitivity indices and glucose control parameters were observed at day 0, 21, and 42 post-transplantation. It should be noted that there were the increasing trends of some insulin sensitivity indices overtime including relative plasma C-peptide level, pancreatic beta-cell function index (HOMA-beta), and insulin resistance index (HOMA-IR) (Fig 4D–4F). However, there was no significant difference between the two groups in each timepoint. The opposite trend was found for the quantitative insulin sensitivity check index (QUICKI) without the significant difference between the two groups for each timepoint (Fig 4G). These may suggest the irrelevance of this phenomena with the transplantation platform.

Further glucose control efficiency was observed using an intraperitoneal glucose tolerance test (IPGTT), and the results illustrated similar trends of glucose control efficiency by the two groups at day 21 and 42 post-transplantation (Fig 4H and 4I). An additional safety study of 10% Pluronic-F127 gel was conducted to ensure the safety of the main reagent used in protocol (S3 and S4 Figs) (S4 and S5 Tables). These results confirmed that the transplantation platform is well-tolerated and has no effects on insulin sensitivity and glucose control.

To characterize the influence of transplantation platform on the transplantation area vascularization, the vessel number along with the histopathology analyses were conducted. The results showed that the transplantation platform could enhance the vessel number around the transplantation site significantly (Fig 4J).

Further histological analysis of the transplantation area found the accumulation of intact blank bead materials within the subcutaneous area of the carrier-bead group at day 21 and 42 post-transplantation suggesting the bead stability under transplantation condition. In addition, an enhanced vascularization of transplantation area of the carrier-bead group was found compared to the control group revealing the efficiency of pocket-retaining gel infusion (Fig 4K). Additional IHC analysis targeting CD31^+^ endothelial cells suggested the presence of CD31^+^ blood vessels at the boundary of subcutaneous pockets and within the transplantation sites of the carrier-bead group (Fig 4L). Since there was a cross-reactivity of the CD31 staining with the bead materials, the structure of histopathology was used to confirm the blood vessel locations.

To further confirm the safety of the transplantation platform, histopathology of insulin-dependent tissues including pancreas, brain, kidney, muscle, and fat were observed by H&E staining together with the gross analysis of transplantation site, brain, pancreas, and kidney (S4 and S5 Figs).

Taken together, the subcutaneous transplantation platform employing a 2-step transplantation protocol is safe and efficient for supporting the IPC bead transplantation study.

### Exploration of the inflammatory cytokine networks in animal receiving carrier-bead transplantation

**Fig 5:**
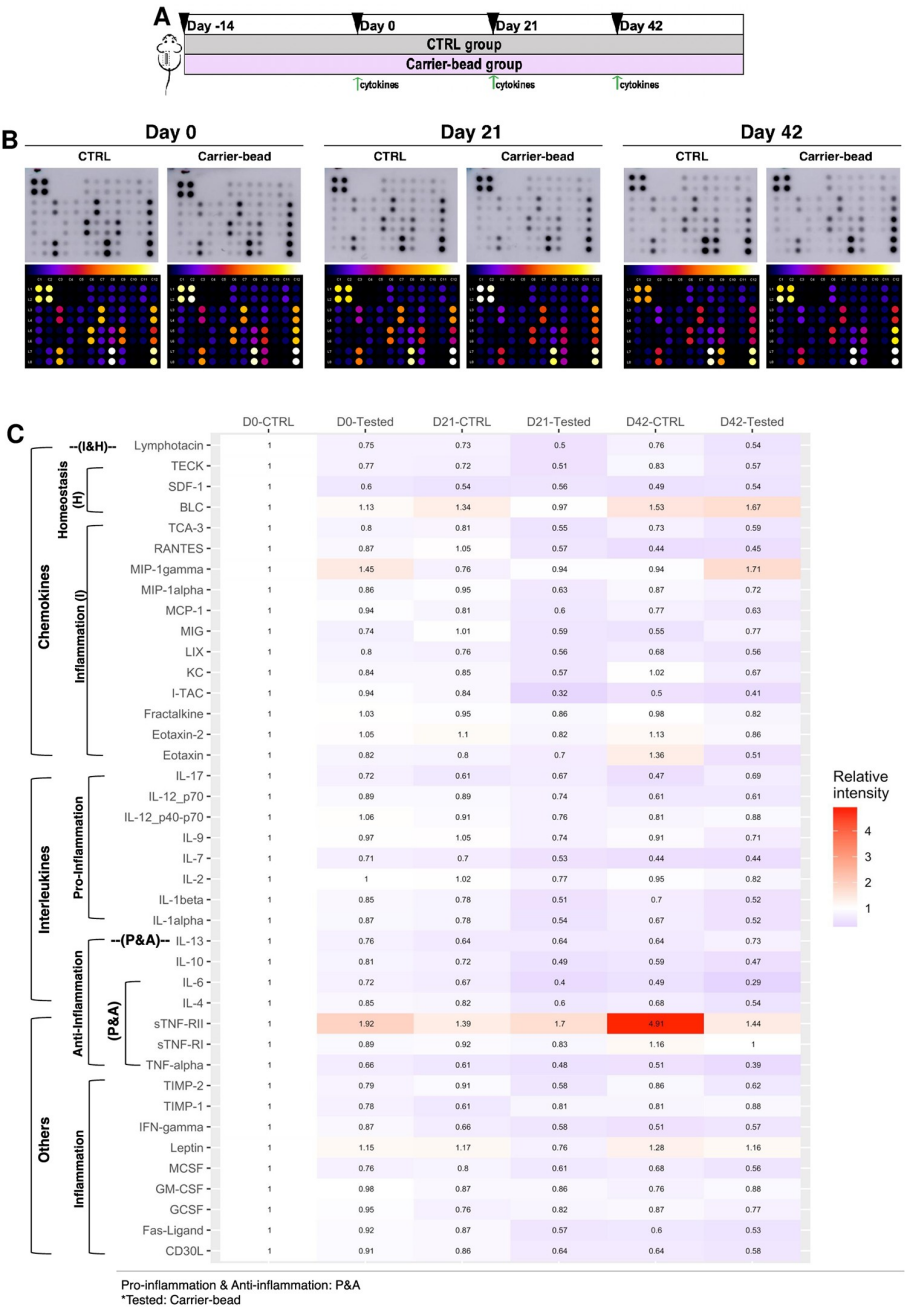
Exploration of the inflammatory cytokine networks in animal receiving carrier-bead transplantation. **(A)** Schematic presentation of inflammatory cytokine network analysis in animal receiving carrier-bead transplantation. **(B)** Cytokine array analysis of inflammatory cytokines represented as clot dots and heat dots of animal receiving carrier-bead transplantation at day 0, 21, and 42 of the study. **(C)** Heatmap and relative levels of 40 inflammatory cytokines of animal receiving carrier-bead transplantation at day 0, 21, and 42 of the study. ***Abbreviations*:** *BLC*: *B lymphocyte chemoattractant*, *GCSF*: *granulocyte colony-stimulating factor*, *GM-CSF*: *granulocyte-macrophage colony-stimulating factor*, *IFN*: *interferon*, *IL*: *interleukin*, *I-TAC*: *interferon-inducible T-cell alpha chemoattractant*, *CXC*: *chemokine*, *KC*: *keratinocyte chemoattractant (chemokine ligand 1)*, *LIX*: *lipopolysaccharide-induced chemokine*, *MCP*: *monocyte chemoattractant protein*, *MCSF*: *macrophage colony-stimulating factor*, *MIG*: *monokine induced by gamma interferon*, *MIP*: *macrophage inflammatory protein*, *RANTES*: *Regulated on activation*, *normal T-cell expressed and secreted*, *SDF*: *stromal cell-derived factor*, *TCA*: *T-cell activation gene*, *TECK*: *thymus expressed chemokine*, *TIMP*: *tissue inhibitor of metalloproteinase*, *TNF(R)*: *tumor necrosis factor (receptor)*, *CD30L*: *CD30 ligand (a member of TNF superfamily)*, *Pos*: *Positive spot*, *Neg*: *negative spot*.

To explore the inflammatory cytokine networks in animals receiving carrier-bead transplantation, a 40-inflammatory cytokine antibody array was employed. The relative plasma cytokine levels of control and carrier-bead groups at day 0, 21, and 42 post-transplantation were benchmarked. Cytokine levels of the control group at day 0 were used as the normalizing values (Fig 5A).

To analyze the data, the clot dots were matched with the manufacture’s array map, then analyzed for heat dots and intensities with ImageJ. The intensities were calculated using the formula $X=(X_{\left(y\right)}-negative)\times \frac{P1}{P_{\left(y\right)}}$ following the manufacturer’s instructions (Figs 5B and S6). A heat map was then generated based on relative intensity using R software, demonstrating the detection of 40 cytokines in both groups.

The results illustrated the presence of 40 cytokines in both control and carrier-bead groups consisting of chemokines, interleukins, and other known inflammatory modulators. It was interesting that the majority of inflammatory cytokines in the carrier-bead group were not surged after the transplantation even at day 21 and 42 compared to CTRL group. There were some minor increasing of cytokines in carrier-bead group (B lymphocyte chemoattractant: BLC, macrophage inflammatory protein 1 gamma: MIP-1 gamma, monokine induced by gamma interferon (MIG), interleukin (IL)-17, IL-13) suggesting a slightly response upon the transplantation period. However, it should be noted that there was a fluctuation of tumor necrosis factor receptor II (sTNF-RII) levels especially in the control group at day 42, without the surging level in the carrier-bead group at the same timepoint. This may suggest the irrelevance of the phenomenon with carrier-bead transplantation.

The results reveal that the subcutaneous transplantation platform contains less inflammatory response and may help minimize the tissue reaction and graft rejection.

### Preliminary subcutaneous IPC-bead transplantation shows positive trends on blood glucose control and sustains survival rate in an induced type I diabetic mouse model

To preliminarily study the potential of the subcutaneous transplantation platform on the IPC delivery, the encapsulated IPC beads were transplanted into an induced type I diabetic mouse model (S7 Fig). In this regard, approximately 100 IPC colonies were encapsulated in each bead which was equivalent to 10% of bead volume (IPC volume = 0.014 mm^3^/colony, bead volume = 14.13 mm^3^/bead). This density was designated as the minimal bead encapsulation capacity.

Twenty-five encapsulated beads were then transplanted into each mouse which was equivalent to 1.4% of mouse body volume (mouse body volume = 25,075.23 mm^3^/25 g mouse) [38]. The estimated ceiling of transplantation volume was set as below 2% of total body volume to ensure blood supply sufficiency.

**Fig 6:**
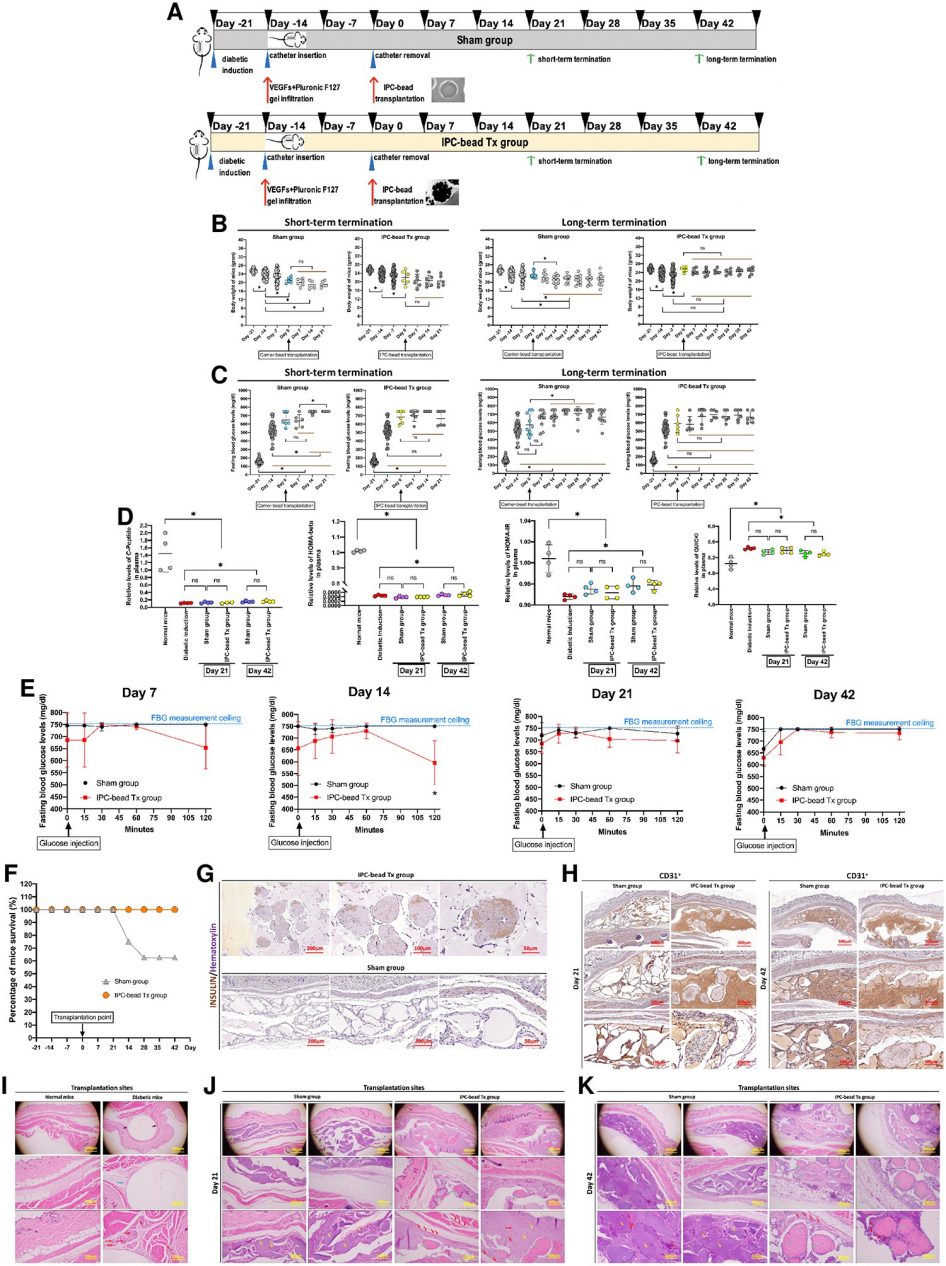
Preliminary subcutaneous IPC-bead transplantation shows positive trends on blood glucose control and sustains survival rate in an induced type I diabetic mouse model. **(A)** Schematic presentation of a preliminary study on subcutaneous IPC-bead transplantation in an induced type I diabetic mouse model. **(B)** Body weight of animals during the study period. **(C)** Fasting blood glucose (FBG) levels of animals during the study period (glucose measurement ceiling = 750 mg/dl). **(D)** Relative C-peptide levels, HOMA-beta, HOMA-IR, and QUICKI indices of animals during the study. **(E)** Intraperitoneal glucose tolerance test (IPGTT) of animals at day 7, 14, 21, and 42 of the study. **(F)** Percentage of animal survival during the study. **(G)** Immunohistochemistry (IHC) analysis for insulin of transplantation site at day 42 of the study. **(H)** IHC analysis for CD31 of transplantation site at day 21 and 42 of the study. **(I)**, **(J)**, **(K)** Histopathological examination of subcutaneous transplantation site at day 0, 21, and 42 of the study using hematoxylin & eosin (H&E) staining. ***Annotations*:** *black arrows*: *inserted catheter sheath*, *orange arrows*: *thick connective tissues*, *blue arrows*: *thin connective tissues*, *green arrows*: *adipose tissues*, *yellow arrows transplanted beads*, *red arrows*: *blood vessels*, *gray arrows*: *IPC colonies*.

After successful induction with a single high-dose streptozotocin (STZ) injection, all type I diabetic mice were randomly allocated into 2 groups receiving encapsulated IPC bead transplantation (IPC-bead Tx) or sham operation (Sham). The transplantation was conducted according to an established 2-step transplantation protocol, then the transplantation outcomes were respectively observed at 21 (short-term study) and 42 days (long-term study) (Fig 6A).

The results showed that some mice lost their body weight after diabetic induction. However, the IPC-bead Tx group showed trend of body weight maintenance after transplantation (Fig 6B). For FBG analysis, all diabetic mice had hyperglycemia after diabetic induction, and the FBG levels of some diabetic mice reached FBG measurement ceiling (750 mg/dl) by 3 weeks after induction suggesting the severity of diabetic conditions. After encapsulated IPC bead transplantation, there was a trend of mild FBG lowering but it was not significantly different compared to the Sham group. This might be due to the severity of diabetic conditions developed in this model (Fig 6C).

Further analyses on insulin sensitivity indices and glucose control capabilities found that the encapsulated IPC bead transplantation with minimal bead encapsulation capacity (10% bead volume) could not restored the levels of fasting plasma C-peptide in both short- and long-term studies. These trends were also found in the analyses of HOMA-beta, HOMA-IR, and QUICKI of the same animal population (Fig 6D). This may suggest that the higher IPC encapsulation volume may be required to resuscitate the basal C-peptide level in diabetic mice.

However, the analysis of glucose control efficiency by IPGTT found that the encapsulated IPC transplantation showed beneficial effects on glucose control efficiency since day 7 post-transplantation (Fig 6E). This might imply the benefits on post-prandial hyperglycemic control. Additional survival rate analysis was conducted and found that the encapsulated IPC transplantation could sustain the survival rate of all diabetic mice until the study ended, while the population of the Sham group was 40% lost (Fig 6F).

Furthermore, the production of insulin by the transplanted encapsulated IPCs was confirmed by the histological analysis of the transplantation area which suggested the bead stability and the presence of insulin within the IPC colonies (Fig 6G). Additional IHC analysis targeting CD31^+^ endothelial cells illustrated the presence of CD31^+^ blood vessels at the transplantation sites (Fig 6H). This was correlated with gross analysis of the subcutaneous transplantation area showing vascularization patterns of the sites (S8 Fig). To further observe the structure of the encapsulated IPC beads and the transplantation sites, histological analyses of the tissues were performed. The results showed the presence of intact encapsulated IPC beads at the transplantation sites with the infiltration of blood vessels throughout the transplantation areas suggesting bead stability and efficient transplantation area preparation (Fig 6I–6K).

To further confirm the safety of the transplantation platform, histopathology of insulin-dependent tissues including pancreas, kidney, brain, muscle, and fat were observed by H&E staining together with the gross analysis of pancreas, brain, kidney, and transplantation site (S9 and S10 Figs). Additional analyses of blood profiles (CBC and blood chemistry) were performed (S6 and S7 Tables). The results suggest the safety of the transplantation platform in terms of tissue analysis and blood parameter analysis.

Taken together, the results support the efficiency and safety of an established subcutaneous IPC transplantation platform in an induced type I diabetic mouse model with the positive trends of blood glucose control as well as the sustained survival rate.

### Inflammatory cytokine network analysis reveals beneficial effects of an encapsulated IPC bead transplantation on an induced type I diabetic mouse model

**Fig 7:**
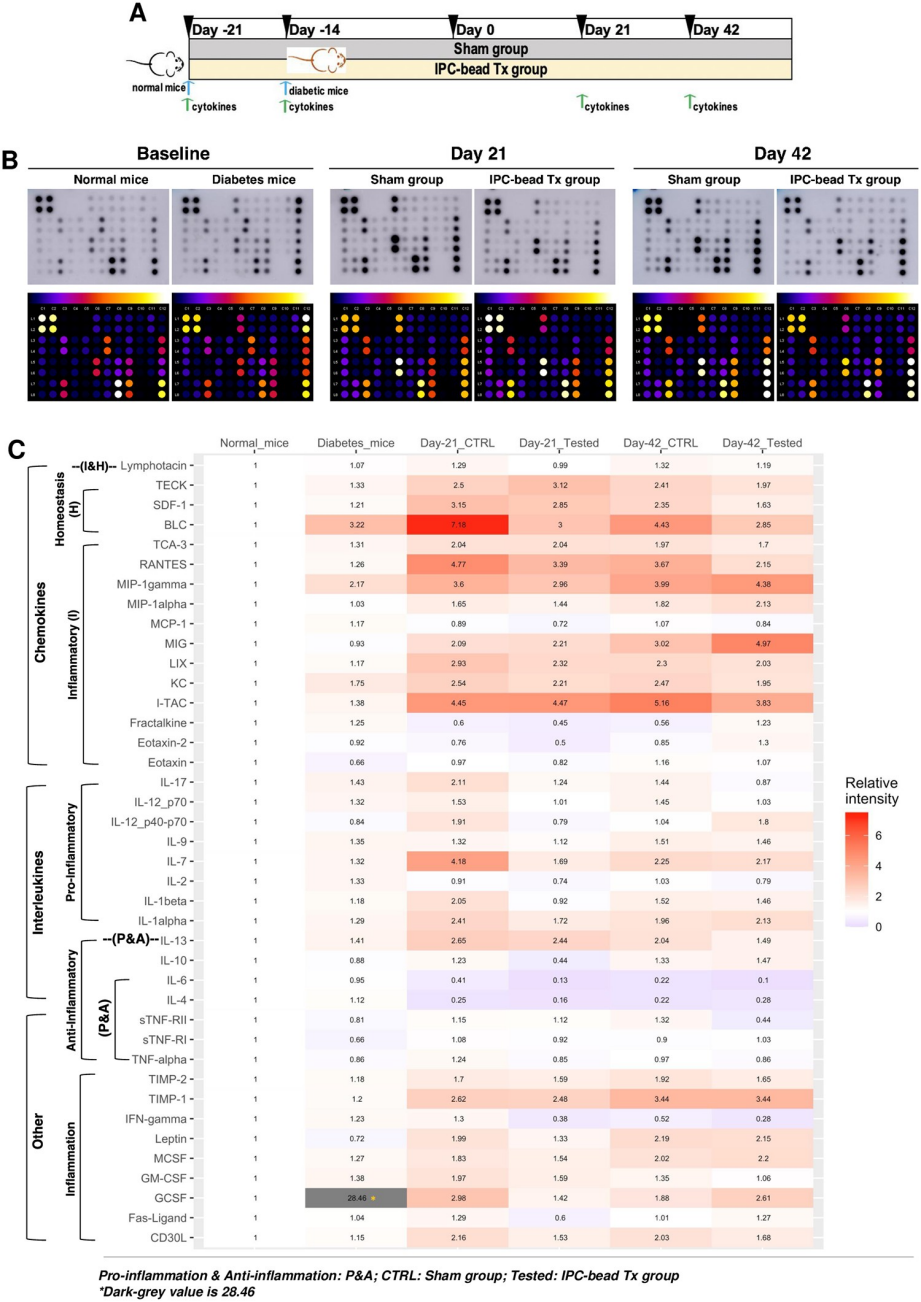
Exploration of the inflammatory cytokine networks of subcutaneous IPC-bead transplantation in an induced type I diabetic mouse model. **(A)** Schematic presentation of inflammatory cytokine network analysis of subcutaneous IPC-bead transplantation in an induced type I diabetic mouse model. **(B)** Cytokine array analysis of inflammatory cytokines represented as clot dots and heat dots of subcutaneous IPC-bead transplantation in an induced type I diabetic mouse model at day 0, 21, and 42 of the study. **(C)** Heatmap and relative levels of 40 inflammatory cytokines of subcutaneous IPC-bead transplantation in an induced type I diabetic mouse model at day 0, 21, and 42 of the study. ***Abbreviations*:** *BLC*: *B lymphocyte chemoattractant*, *GCSF*: *granulocyte colony-stimulating factor*, *GM-CSF*: *granulocyte-macrophage colony-stimulating factor*, *IFN*: *interferon*, *IL*: *interleukin*, *I-TAC*: *interferon-inducible T-cell alpha chemoattractant*, *CXC*: *chemokine*, *KC*: *keratinocyte chemoattractant (chemokine ligand 1)*, *LIX*: *lipopolysaccharide-induced chemokine*, *MCP*: *monocyte chemoattractant protein*, *MCSF*: *macrophage colony-stimulating factor*, *MIG*: *monokine induced by gamma interferon*, *MIP*: *macrophage inflammatory protein*, *RANTES*: *Regulated on activation*, *normal T-cell expressed and secreted*, *SDF*: *stromal cell-derived factor*, *TCA*: *T-cell activation gene*, *TECK*: *thymus expressed chemokine*, *TIMP*: *tissue inhibitor of metalloproteinase*, *TNF(R)*: *tumor necrosis factor (receptor)*, *CD30L*: *CD30 ligand (a member of TNF superfamily)*, *Pos*: *Positive spot*, *Neg*: *negative spot*.

To explore the inflammatory cytokine networks in animals receiving encapsulated IPC bead transplantation, a 40-inflammatory cytokine antibody array analysis was conducted. The relative plasma cytokine levels of normal mice, diabetic mice, and diabetic mice receiving encapsulated IPC bead transplantation or sham operation at day 21 and 42 post-transplantation were benchmarked. Cytokine levels of normal mice were used as the normalizing values (Fig 7A). To analyze the data, the clot dots were matched with the manufacture’s array map, then analyzed for heat dots and intensities with ImageJ. The intensities were calculated following the manufacturer’s instructions (Figs 7B and S6). A heat map was then generated based on relative intensity using R software, demonstrating the detection of 40 cytokines in animals.

The results illustrated the presence of 40 cytokines in all experimental groups consisting of chemokines, interleukins, and other known inflammatory modulators. It should be noted that the majority of inflammatory cytokines in diabetic mice were increased compared to normal mice, especially BCL, MIP-1 gamma, and granulocyte-colony stimulating factor (GCSF). This might suggest the systemic inflammatory condition in diabetic mice.

After encapsulated IPC bead transplantation, most of cytokine levels of diabetic mice receiving encapsulated IPC bead transplantation were declined compared with sham group at day 21 and 42 post-transplantation, respectively (Fig 7C). The results imply that the encapsulated IPC bead transplantation might alleviate the systemic inflammatory conditions of diabetic mice and has less immunological response or graft rejection.

## Discussion

Loss of pancreatic beta-cells in type I DM causes insulin depletion, hyperglycemia, dysregulation of glucose homeostasis, and eventually death in uncontrolled diabetic patients and animals [39–41]. It has been shown that cadaveric pancreatic islet transplantation successfully restores the normoglycemia without additional exogenous insulin administration according to Edmonton protocol studies in human [2,4,42]. However, the key interventions required for the successful transplantation outcomes were rigid, especially islet capacity, transplantation islet amount, islet biocompatibility, islet donor availability, and long-term immunosuppressant administration in recipients [4,43,44]. These factors lower the success rate and treatment availability for public.

To overcome these problems, an application of stem-based insulin-producing cell (IPC) production has been introduced with the promising efficiency of an induced pluripotent stem cells (iPSCs) [17,21,43,45,46]. This has paved the way for the potential application of patient-specific autologous stem cell-based therapy for degenerative diseases and autoimmune disorders due to the less requirement on recipient immunosuppression [47,48]. In addition, an efficient transplantation platform that can minimize the immunological responses may benefit the clinical application of allogenic stem cell-based treatments [35,49]. In this study, the induced pluripotent stem cells (iPSCs) generated from mouse gingival fibroblasts (mGFs) of C57BL/6J mice (mGF-iPSCs) were used as a cell resource for generating the transplantable insulin-producing cells (IPCs). The mGF-iPSC-derived IPCs (mGF-iPSC-IPCs) were then transplanted into the C57BL/6NJcl mice after diabetic induction by streptozotocin as an allogeneic transplantation model.

Since the first clinical study in autologous iPSC-derived specific cell transplantation for treating an age-related macular degeneration in 2014 [50], several clinical studies on the autologous or allogeneic iPSC-derived cell transplantation model have been conducted suggesting the potential application in human and veterinary medicine [51,52]; ClinicalTrials.gov: NCT02464956, NCT04339764, NCT05566600, NCT05616338, NCT01943383.

In this study, the mGF-iPSCs generated from gingival fibroblasts of C57BL/6J mice were used as the starting cells for generating the transplantable IPCs using in the transplantation model establishment [36]. To efficiently induce the cells toward pancreatic lineage, the series of 6-step induction protocol were established and validated. We found that the low attachment culture vessels were essential for the generation of embryoid bodies and the subsequent pancreatic colonies which was correlated with our previous studies [5–8]. It should be noted that the treatment of Notch inhibitor (DAPT) and GLP-1 during the induction process could enhance the basal relative C-peptide secretion by the IPCs suggesting the maturation of the cells. This result was correlated with our previous reports suggesting the relevance of Notch signaling on the pancreatic lineage commitment and IPC maturation [5,7,53,54]. Recent studies showed the success on autologous IPC generation from human iPSCs derived from type I DM and type II DM patients suggesting the potential application of autologous iPSC-derived specific cells in further clinical trials [21,55].

Regarding the strategy for setting up the practical clinical protocol for tissue regeneration or transplantation, the potential starting cell resources are the key factors influencing the success of the protocol. In this study, the model of gingival fibroblasts was evaluated for its potential as starting tissue for the iPSC generation and the production of transplantable IPCs.

It has been shown that dental tissues serve as the potential cell resources for regenerative treatment due to their properties comprising tissue accessibility, availability, and plasticity [5,7,8,30,56]. Compared to other cell resources, isolating cells from gingival tissue is less invasive than those protocols for bone marrow and adipose tissue collections [7]. This information supports the potential of gingival fibroblast-derived cells’ application on tissue regenerative therapy.

In this study, the IPC induction platform was strategically set up based on IPC production efficiency focusing on IPC colony morphology, functional property based on glucose-stimulated C-peptide secretion (GSCS) assay, pancreatic mRNA marker expression along with long-term maintenance of the IPCs regarding cell viability study [8]. The efficient induction protocol will provide the robust and practical production of IPCs for further clinical applications.

In this regard, types of cell culture containers for propagating embryoid bodies and IPCs were considered as an essential initial step of the protocol establishment. Our results suggested that the ultralow attachment or Petri dish vessels were required for the generation of IPCs. Additionally, the modification of induction molecules/steps for enhancing the protocol efficiency is crucial, especially in the aspects of IPC colony production yield, maturation of IPCs regarding pancreatic lineage, functional property of IPCs regarding glucose sensing and glucose-dependent insulin secretion.

Our studies and previous reports found that applying of potential signaling molecules governing Notch signaling and fibroblast growth factor (FGF) could enhance the pancreatic differentiation commitment of the stem cells and the early pancreatic progenitors [5,7,57]. The treatment with glucagon-like peptide (GLP)-1 could benefit the maturation state of the IPCs and the functional property regarding the glucose-responsive insulin secretion [7,8]. In this study, combinations of growth factors and signaling molecules were used to enhance the production yield and the functional property of the IPCs as represented in the final protocol used for IPC generation in transplantation model.

Additionally, the IPCs generated in this study could survive in long-term maintenance with an efficient secretion of C-peptide upon the stimulation which suggests the flexibility for use in expanded production batch. Along with the validated IPC encapsulation protocol, the insulin-secretion capacity and transplantation potential were preserved as mentioned in our previous studies [8,30]. In general, cell encapsulation prevents or lowers immune responses by recipients and may support bio-transport of oxygen, nutrients, and metabolic wastes within the transplantation sites [58–60]. Other factors influencing the success of IPC transplantation are the suitable transplantation site preparation and cell transplantation protocols. To achieve this goal, a 2-step subcutaneous transplantation protocol was developed in this study, comprising 1) vascularized subcutaneous pocket formation and 2) encapsulated IPC bead transplantation. We formulated the subcutaneous pocket retaining gel containing the sterile mixture of VEGF-165 and Pluronic-F127 which helps enhance vascularization and maintain subcutaneous transplantation space effectively.

It has been found that VEGF-165 is the main splicing variants of VEFG-A and triggers biological activities via the binding with membrane receptors including vascular endothelial growth factor receptor (VEGFR)-1, VEGFR-2, Neuropilin (NRP)-1, and NRP-2 [61]. The study in human melanoma cell line found that the binding of VEGF-165 to VEGFR-1 enhances cell migration in both chemotactic and chemokinetic patterns, but not the cell proliferation. This phenomenon employs a phosphatidylinositol-3 kinase (PI3K)/Akt pathway [62]. The main effects of VEGF-165 on vascularization may be triggered by VEGFR-2 signaling that causes endothelial cell migration and proliferation [61,63]. Recent study has suggested the rejuvenating effect of VEGF-A on an aged human skin transplant in xenotransplantation model using SCID/beige mice [64].

In addition, the Pluronic-F127 used in this transplantation model could facilitate the delivery of active substances to the surrounding tissues as seen in the previous study that employed Pluronic-F127 to deliver the glycogen synthase kinase 3 beta (GSK3β) inhibitor (6-bromoindirubin-3′-oxime) and mitigate inflammatory responses effectively in ischemic stroke animal model [65]. Further studies have shown the beneficial properties of Pluronic-F127 as a reservoir for high-dose stem cell loading and bioink formulation [66,67].

To primarily observe the potential of the subcutaneous transplantation platform, the preliminary study of encapsulated IPC bead transplantation was conducted in an induced type I diabetic mouse model. The encapsulated IPC volume was set at 10% of bead volume which is designated as the minimal encapsulation capacity. The volume of transplanted bead per animal was set at below 2% of animal body volume to ensure blood supply sufficiency [38]. With this IPC dosage, the type I diabetic mice could regain their body weight and showed an improved glucose control efficiency as measured by an IPGGT assay suggesting the benefits on post-prandial hyperglycemic control. In addition, the encapsulated IPC transplantation could sustain the survival rate of all diabetic mice, while the sham group showed 40% mortality.

It has been proposed that the promising IPC transplantation for both human and veterinary practices require a less invasive technique with the beneficial properties on IPC stability, sufficient oxygen and nutrient permeability, and biocompatibility [12,68]. By this means the subcutaneous transplantation of the macro- or micro-encapsulated IPCs is a potential platform for clinical application [69–73]. In general, the subcutaneous transplantation is considered as a device-less and painless protocol with minimal invasive to internal organs [35,74,75]. Several subcutaneous transplantation studies have revealed the enhanced vascularization and well-permeated oxygen and nutrients within the transplantation sites which enhance the success rate of IPC transplantation [76,77]. The strategies that play crucial roles on subcutaneous IPC transplantation rely on the pre-transplantation preparation of the transplantation site, the preparation of matured IPC with suitable cell coating or encapsulation, and the post-transplantation maintenance and follow up [12,68]. According to the studies on pancreatic islet transplantation, the recipients require life-long administration of immunomodulators or immunosuppressants to ensure the graft success rate. However, most of immunosuppressive drugs used clinically have serious adverse events (AEs) and less patient compliance [78,79].

In this study, the antibody array was used to explore the dynamic of systemic inflammatory cytokines upon the encapsulated IPC bead transplantation in diabetic mice. It was interesting that most of inflammatory cytokines were surged in diabetic mice, particularly BCL, MIP-1, and GCSF. These findings revealed the systemic inflammatory condition in diabetic mice. After encapsulated IPC bead transplantation, most of cytokine levels declined. The safety studies regarding gross and histopathological analyses and blood profiling (CBC and blood chemistry) suggested the safety of transplantation platform.

Streptozotocin (STZ) is a diabetogenic glucose analogue with cytotoxic effects on mammalian cells due to the DNA fragmentation triggered by the methylnitrosourea moiety in the STZ structure [80] along with an increased production of nitric oxide (NO) and reactive oxygen species (ROS) causing DNA alkylation and oxidative stress [81,82]. It has been showed that the pancreatic cytotoxicity of STZ is dose-dependent and selective to pancreatic beta-cells by glucose transporter type 2 (GLUT-2) uptake [83,84].

However, hepatocytes and renal tubular cells also express GLUT-2 making them susceptible to STZ toxicity [85,86]. To avoid the non-specific cytotoxic effects of STZ in diabetic animal models, the selected dose range of STZ was used for scoping the effect of STZ to pancreatic beta-cells. The dose of STZ for mice ranges from 175–200 mg/kg intraperitoneal (IP) or intravenous (IV) [87]. In this study, the single intraperitoneal administration of STZ at 180 mg/kg was employed. To observe the non-specific cytotoxic effects of STZ on liver and kidney, the blood chemistry profile and histopathological examinations of the tissues were processed and found only an increased alanine aminotransferase (ALT) of the non-treated diabetic animals at day 21 of the long-term study phase. This finding may be related to the in-direct cytotoxicity of STZ on hepatic parenchyma [88].

According to the transplantation study in diabetic mouse model, the encapsulated mGF-iPSC-IPCs could last for 42 days as seen in long-term study (42 days) with intact encapsulated IPC bead morphology at transplantation sites along with the presence of insulin within the IPC colonies as analyzed by IHC staining. Additionally, the pre-transplantation analyses of mGF-iPSC-IPCs found that the colony encapsulation could sustain the IPC morphology and functional properties along with the expression of insulin until day 53 of the maintenance period. As per the information acquired from the study, we proposed that the encapsulated mGF-iPSC-IPCs could last for at least 53 days. However, to clarify the half-life of the encapsulated IPCs, the long-term and continuous study of the IPC transplantation on diabetic animal model should be further conducted.

Regarding the comparison between IPC transplant and conventional islet transplant, it should be noted that both protocols aim for effective blood glucose control without exogenous insulin administration. The Edmonton protocol was a successful clinical trial by employing the cadaveric islet transplantation. However, due to the administration of immunosuppressants and the shortage of donors, this protocol seems to have a burden for widely clinical application [2]. To address these issues, stem cell-derived IPCs have been introduced for solving the problem on islet donor shortage, and the study on transplantation platform was set as a key component for achieving a feasible clinical application [7,8]. Hence, the knowledge from both platforms is fulfilling each other to achieve an ultimate goal on effective diabetes control.

In this study, the effects of encapsulated IPC transplantation on surrounding tissues were observed by gross and histological analyses at day 21 and 42 post-transplantation. It has been shown that the transplantation-surrounding tissues were infiltrated with vascular networks along with the connective tissues. There was no remarkable finding of tissue inflammation post-transplantation. Additional analysis on systemic immunological responses by inflammatory cytokine antibody array also confirmed that the encapsulated IPC bead transplantation did not trigger systemic immunological response suggesting the immunological compatibility between the IPC graft and host.

Previous publications proposed that immunological response and graft rejection might be the crucial factors predicting the success of islet transplantation in type 1 diabetic patients [89,90]. Several approaches have been introduced to overcome the immunological burdens upon islet or IPC transplantations, for example, cell- and cytokine-based therapies on immune cell recruitment, cell reprogramming to avoid immune cell attack, and macro- or micro-encapsulation to avoid immune cell interaction [12,90]. Hence the practical and cost-effective protocol reflects the potential application in clinical practice, the IPC transplantation using macro-encapsulation technique used in this study may be the potential solution for further clinical application in both human and veterinary practices.

To emphasize on the potential challenges of the study, the crucial factors influencing the clinical IPC transplantation are of concern, especially the efficiency of transplanted IPCs on long-term blood glucose control and the IPC survival period to estimate the IPC lifespan and the timeline for re-transplantation. In this study, our *in vitro* studies showed that the encapsulation of IPCs and the maintenance protocol could sustain the IPC survival for more than 6 weeks. However, to ensure the clinical efficacy of the platform in diabetic patients, it may be necessary for IPCs to survive and efficiently control blood glucose levels for at least six months in clinical applications [91,92]. Our future study will be focused on long-term control of blood glucose in diabetic models and patients.

This study will provide a fundamental platform for further research on IPC production and transplantation in human and veterinary diabetic patients. The IPC induction protocol and encapsulation platform will be one of the cell protection approaches for transplantation as well as the subcutaneous transplantation protocols that will supports a less immunogenic cell transplantation for clinical applications. In essential, the cost-effective clinical protocol will provide a practical application of future stem cell-based therapy for diabetic patients in both human and veterinary practices.

To accomplish the clinical application of stem cell therapy on diabetes control and treatment, the pre-clinical evaluation on safety and effectiveness of the platform should be fully validated to ensure the successful implementation in human and veterinary practices. Further studies on primate models and target animal models are crucial for stepping forward to clinical studies in human and veterinary practices, respectively.

## Conclusion

In this study, the standard protocol for generating IPCs from mGF-iPSCs was successfully established, and the macro-encapsulation using double coating strategy was verified. An established 2-step transplantation protocol, comprising 1) vascularized subcutaneous pocket formation and 2) encapsulated IPC bead transplantation, was then validated in the induced diabetic animal model which was an artificial experiment. The results suggested the potential application of the transplantation protocol regarding its safety and efficiency. Further clinical studies are required for confirming an effectiveness in human and veterinary applications.

## Supporting information

S1 Fig Batch characterization of mGF-derived iPSCs (mGF-iPSCs) and validation of embryoid body production protocol. **(A)** Expression of stemness mRNA markers (*Nanog*, *Rex1*, *Oct4*, *Sox2*, and *Ssea1*) by mGF-iPSCs using RT-qPCR. **(B)** Alkaline phosphatase (ALP) staining of mGF-iPSC colonies. **(C)** Glucose-stimulated C-peptide secretion (GSCS) analysis of day-4 mGF-iPSC embryoid bodies. **(D)** Morphological appearance of day-4 mGF-iPSC colonies. **(E)** Morphological appearance of day-4 mGF-iPSC embryoid bodies generated in different low-attachment containers.
(TIF)

S2 Fig Subcutaneous pocket formation procedures. Reprinted from “Materials for supplementary figures: Establishment of subcutaneous transplantation platform for delivering induced pluripotent stem cell-derived insulin-producing cells” under a CC BY license, with permission from Chenphop Sawangmake, original copyright 2024.
(TIF)

S3 Fig A 7-day validation of subcutaneous pocket formation using 10% Pluronic acid in normal mice. **(A)** Schematic presentation of a 7-day validation of subcutaneous pocket formation using 10% Pluronic acid in normal mice. **(B)** Body weight of animals during the study period. **(C)** Histopathological examination of subcutaneous pocket formation site at day 7 of the study using hematoxylin & eosin (H&E) staining. **(D)** Gross examination of animals and vital organs (pancreas, kidney, spleen, brain, liver, and subcutaneous transplantation site) of animals at day 7 of the study. Reprinted from “Materials for supplementary figures: Establishment of subcutaneous transplantation platform for delivering induced pluripotent stem cell-derived insulin-producing cells” under a CC BY license, with permission from Chenphop Sawangmake, original copyright 2024.
(TIF)

S4 Fig Histopathological examination of insulin-dependent tissues of animals undergone subcutaneous IPC transplantation platform establishment. **(A)**, **(B)**, **(C)**, **(D)**, **(E)** Histopathological examination of insulin-dependent tissues (pancreas, hippocampus, kidney, muscle, and fat) of animals undergone subcutaneous IPC transplantation platform establishment at day 0, 21, and 42 of the study using hematoxylin & eosin (H&E) staining.
(TIF)

S5 Fig Gross examination of subcutaneous transplantation site and insulin-dependent tissues of animals undergone subcutaneous IPC transplantation platform establishment. Reprinted from “Materials for supplementary figures: Establishment of subcutaneous transplantation platform for delivering induced pluripotent stem cell-derived insulin-producing cells” under a CC BY license, with permission from Chenphop Sawangmake, original copyright 2024.
(TIF)

S6 Fig Cytokine array map. (TIF)

S7 Fig Subcutaneous encapsulated IPC-bead transplantation procedure. Reprinted from “Materials for supplementary figures: Establishment of subcutaneous transplantation platform for delivering induced pluripotent stem cell-derived insulin-producing cells” under a CC BY license, with permission from Chenphop Sawangmake, original copyright 2024.
(TIF)

S8 Fig Gross examination of blood vessel generation at the subcutaneous area of transplantation pocket formation site. Reprinted from “Materials for supplementary figures: Establishment of subcutaneous transplantation platform for delivering induced pluripotent stem cell-derived insulin-producing cells” under a CC BY license, with permission from Chenphop Sawangmake, original copyright 2024.
(TIF)

S9 Fig Histopathological examination of insulin-dependent tissues of animals undergone subcutaneous IPC-bead transplantation in an induced type I diabetic mouse model. **(A)**, **(B)**, **(C)**, **(D)**, **(E)** Histopathological examination of insulin-dependent tissues (pancreas, kidney, hippocampus, muscle, and fat) of animals undergone subcutaneous IPC-bead transplantation in an induced type I diabetic mouse model at day 0, 21, and 42 of the study using hematoxylin & eosin (H&E) staining. ***Annotations*:** *black asterisks*: *interlobular duct*, *yellow asterisks*: *pancreatic parenchyma*, *blue asterisks*: *regenerative islets*, *bright blue arrows*: *glomeruli and interstitial areas*, *bright green*: *tuberous*, *short red arrows*: *dentate gyrus*, *short yellow arrows*: *CA2*, *CA3 areas*, *bright blue asterisks*: *adipose cells*.
(TIF)

S10 Fig Gross examination of subcutaneous transplantation site and insulin-dependent tissues of animals undergone subcutaneous IPC-bead transplantation in an induced type I diabetic mouse model. Reprinted from “Materials for supplementary figures: Establishment of subcutaneous transplantation platform for delivering induced pluripotent stem cell-derived insulin-producing cells” under a CC BY license, with permission from Chenphop Sawangmake, original copyright 2024.
(TIF)

S1 Table Primer sequences. (PDF)

S2 Table Complete blood count parameters of animals undergone subcutaneous IPC transplantation platform establishment. (PDF)

S3 Table Blood chemistry parameters of animals undergone subcutaneous IPC transplantation platform establishment. (PDF)

S4 Table Complete blood count parameters of a 7-day validation of subcutaneous pocket formation using 10% Pluronic acid in normal mice. (PDF)

S5 Table Blood chemistry parameters of a 7-day validation of subcutaneous pocket formation using 10% Pluronic acid in normal mice. (PDF)

S6 Table Complete blood count parameters of animals undergone subcutaneous IPC-bead transplantation in an induced type I diabetic mouse model. (PDF)

S7 Table Blood chemistry parameters of animals undergone subcutaneous IPC-bead transplantation in an induced type I diabetic mouse model. (PDF)
