# Supplementary figures and images for: Establishment of subcutaneous transplantation platform for delivering induced pluripotent stem cell-derived insulin-producing cells
Source: PLoS One. 2025 Jan 30;20(1):e0318204. doi: 10.1371/journal.pone.0318204 (PMC11781742; doi:10.1371/journal.pone.0318204)

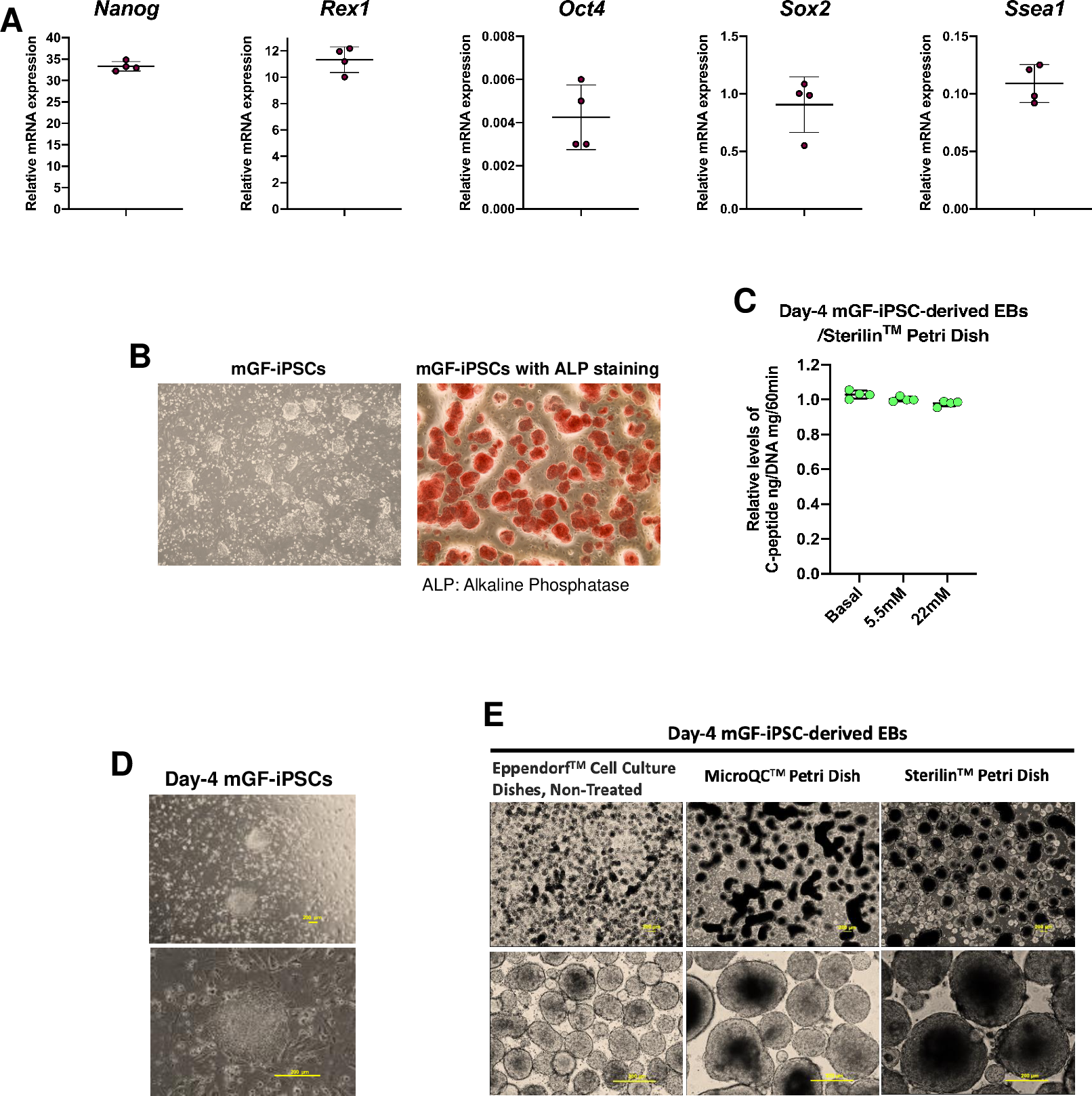

Supplement: S1 Fig — (A) Expression of stemness mRNA markers (Nanog, Rex1, Oct4, Sox2, and Ssea1) by mGF-iPSCs using RT-qPCR. (B) Alkaline phosphatase (ALP) staining of mGF-iPSC colonies. (C) Glucose-stimulated C-peptide secretion (GSCS) analysis of day-4 mGF-iPSC embryoid bodies. (D) Morphological appearance of day-4 mGF-iPSC colonies. (E) Morphological appearance of day-4 mGF-iPSC embryoid bodies generated in different low-attachment containers. (TIF) [file pone.0318204.s001.tif]

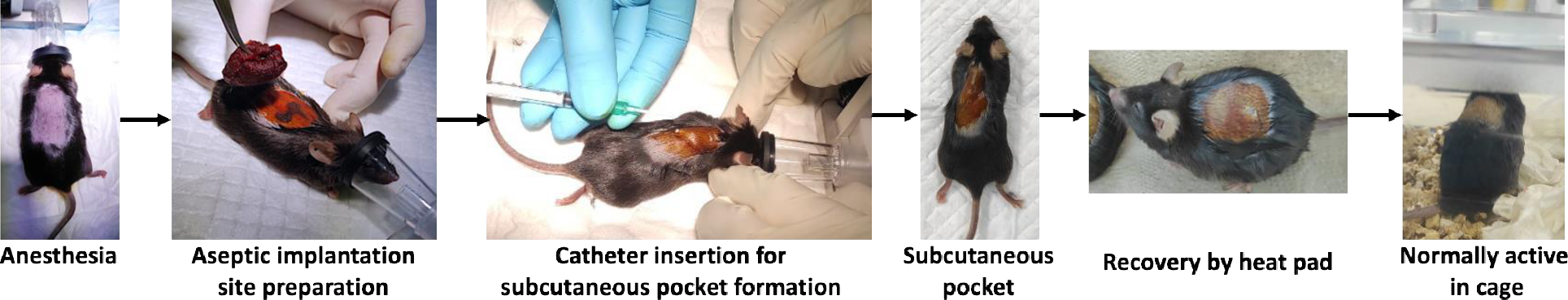

Supplement: S2 Fig — Reprinted from “Materials for supplementary figures: Establishment of subcutaneous transplantation platform for delivering induced pluripotent stem cell-derived insulin-producing cells” under a CC BY license, with permission from Chenphop Sawangmake, original copyright 2024. (TIF) [file pone.0318204.s002.tif]

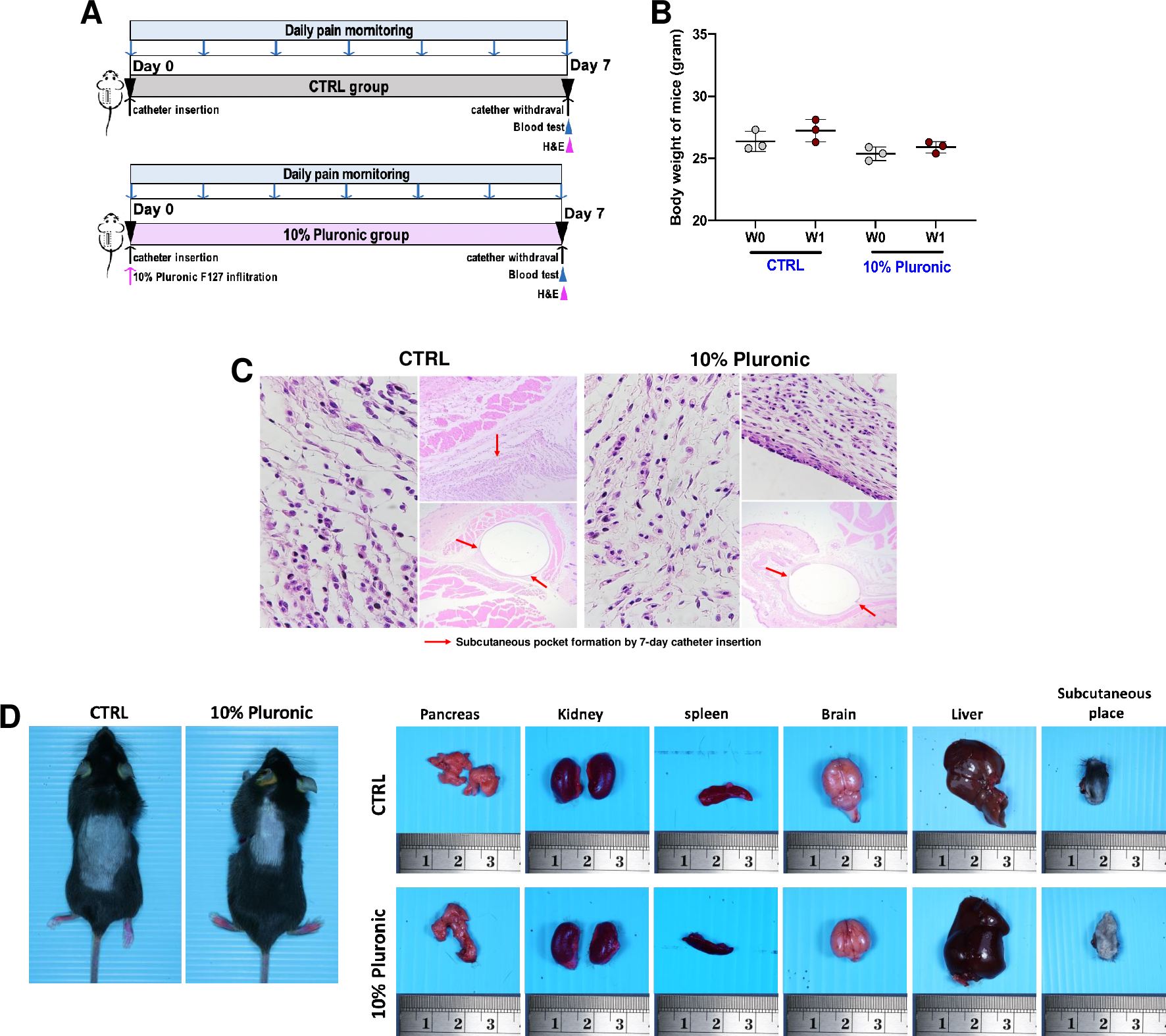

Supplement: S3 Fig — (A) Schematic presentation of a 7-day validation of subcutaneous pocket formation using 10% Pluronic acid in normal mice. (B) Body weight of animals during the study period. (C) Histopathological examination of subcutaneous pocket formation site at day 7 of the study using hematoxylin & eosin (H&E) staining. (D) Gross examination of animals and vital organs (pancreas, kidney, spleen, brain, liver, and subcutaneous transplantation site) of animals at day 7 of the study. Reprinted from “Materials for supplementary figures: Establishment of subcutaneous transplantation platform for delivering induced pluripotent stem cell-derived insulin-producing cells” under a CC BY license, with permission from Chenphop Sawangmake, original copyright 2024. (TIF) [file pone.0318204.s003.tif]

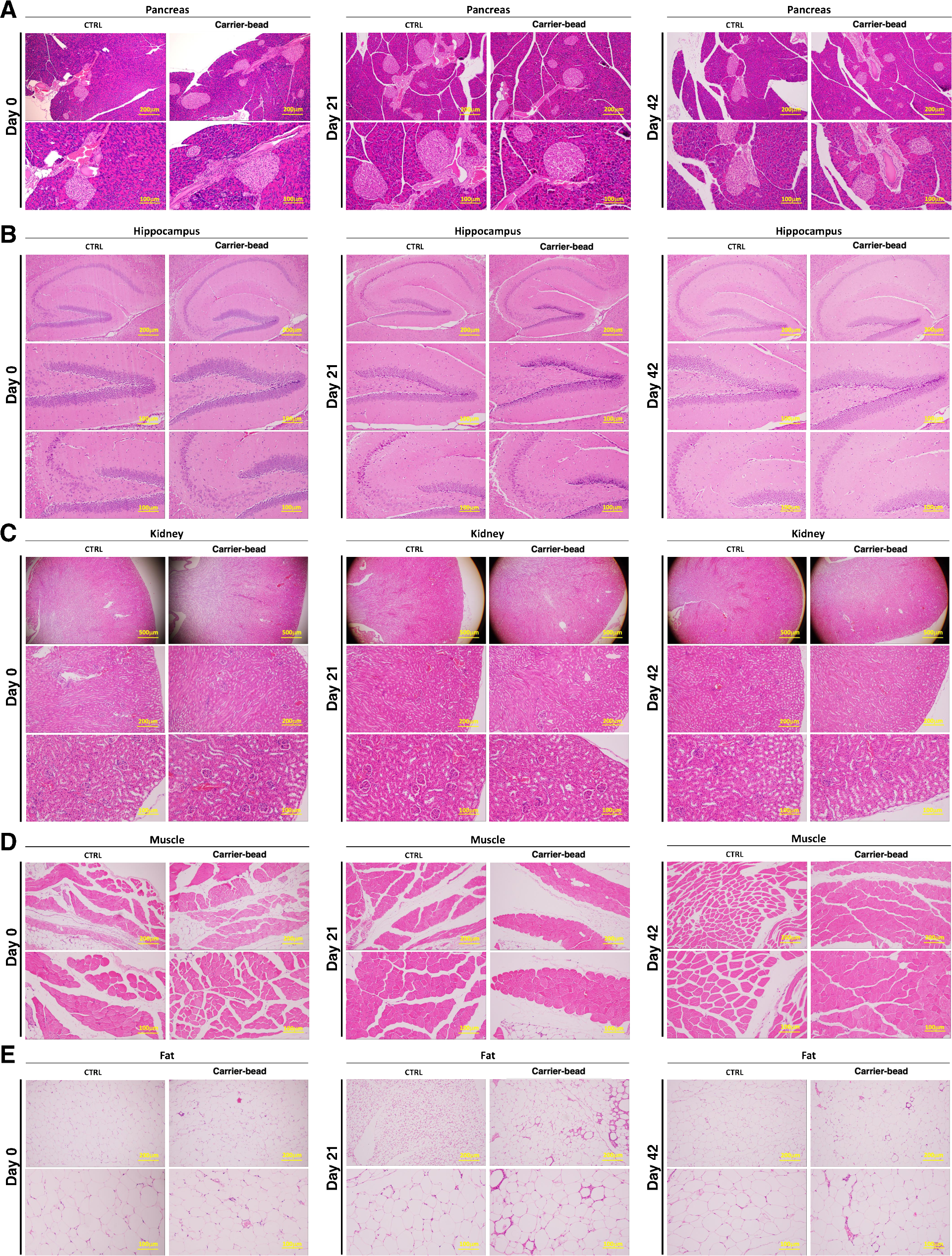

Supplement: S4 Fig — (A), (B), (C), (D), (E) Histopathological examination of insulin-dependent tissues (pancreas, hippocampus, kidney, muscle, and fat) of animals undergone subcutaneous IPC transplantation platform establishment at day 0, 21, and 42 of the study using hematoxylin & eosin (H&E) staining. (TIF) [file pone.0318204.s004.tif]

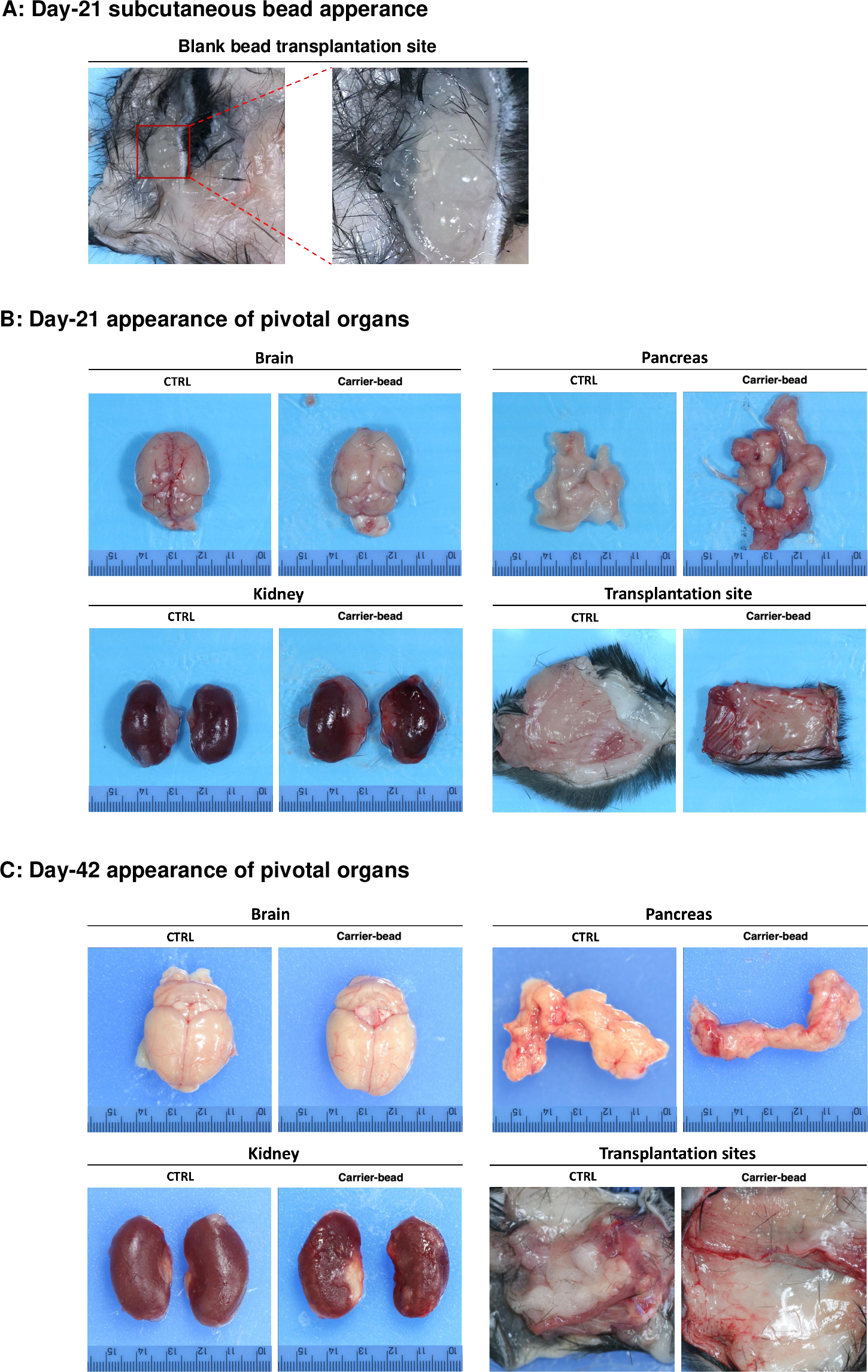

Supplement: S5 Fig — Reprinted from “Materials for supplementary figures: Establishment of subcutaneous transplantation platform for delivering induced pluripotent stem cell-derived insulin-producing cells” under a CC BY license, with permission from Chenphop Sawangmake, original copyright 2024. (TIF) [file pone.0318204.s005.tif]

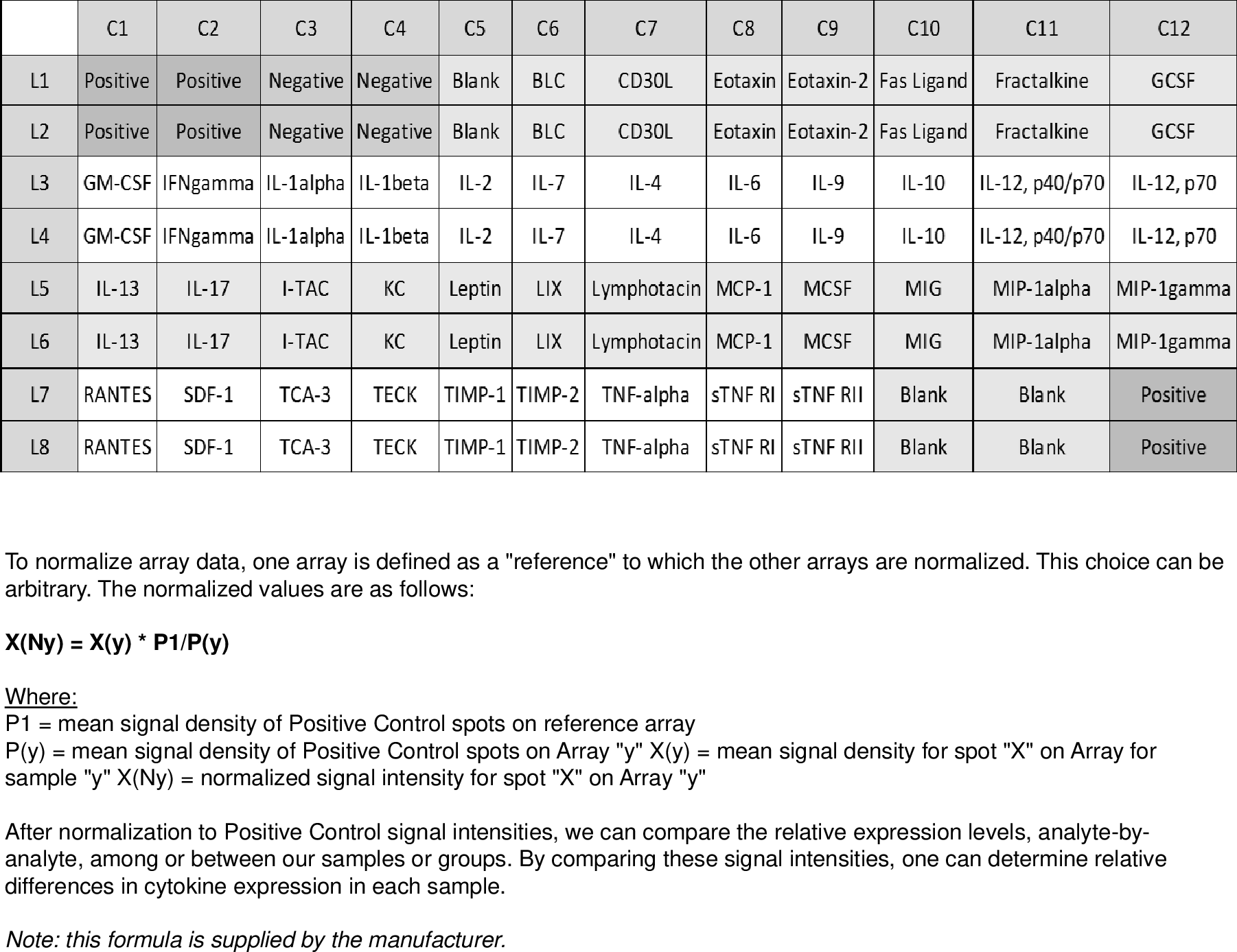

Supplement: S6 Fig — (TIF) [file pone.0318204.s006.tif]

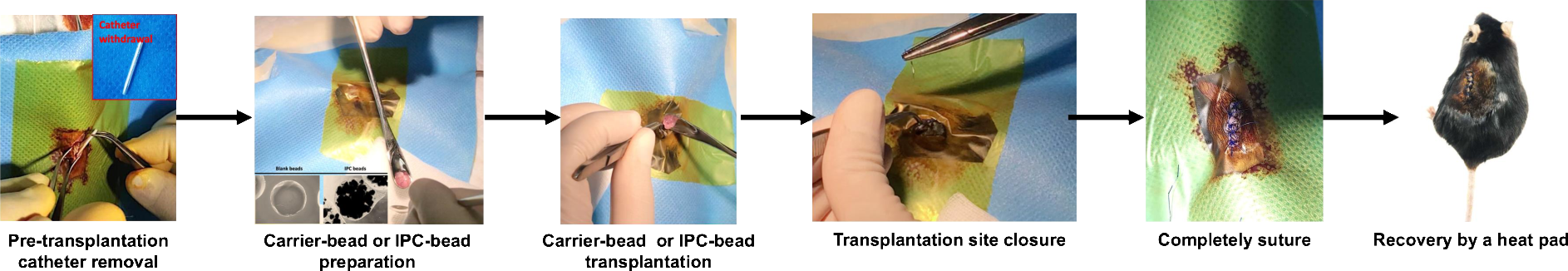

Supplement: S7 Fig — Reprinted from “Materials for supplementary figures: Establishment of subcutaneous transplantation platform for delivering induced pluripotent stem cell-derived insulin-producing cells” under a CC BY license, with permission from Chenphop Sawangmake, original copyright 2024. (TIF) [file pone.0318204.s007.tif]

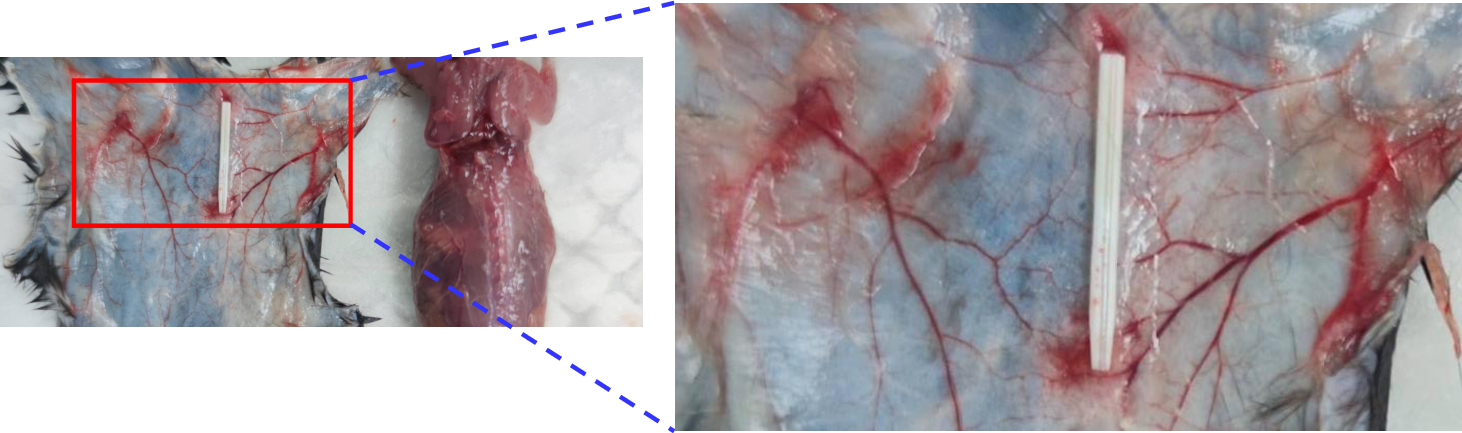

Supplement: S8 Fig — Reprinted from “Materials for supplementary figures: Establishment of subcutaneous transplantation platform for delivering induced pluripotent stem cell-derived insulin-producing cells” under a CC BY license, with permission from Chenphop Sawangmake, original copyright 2024. (TIF) [file pone.0318204.s008.tif]

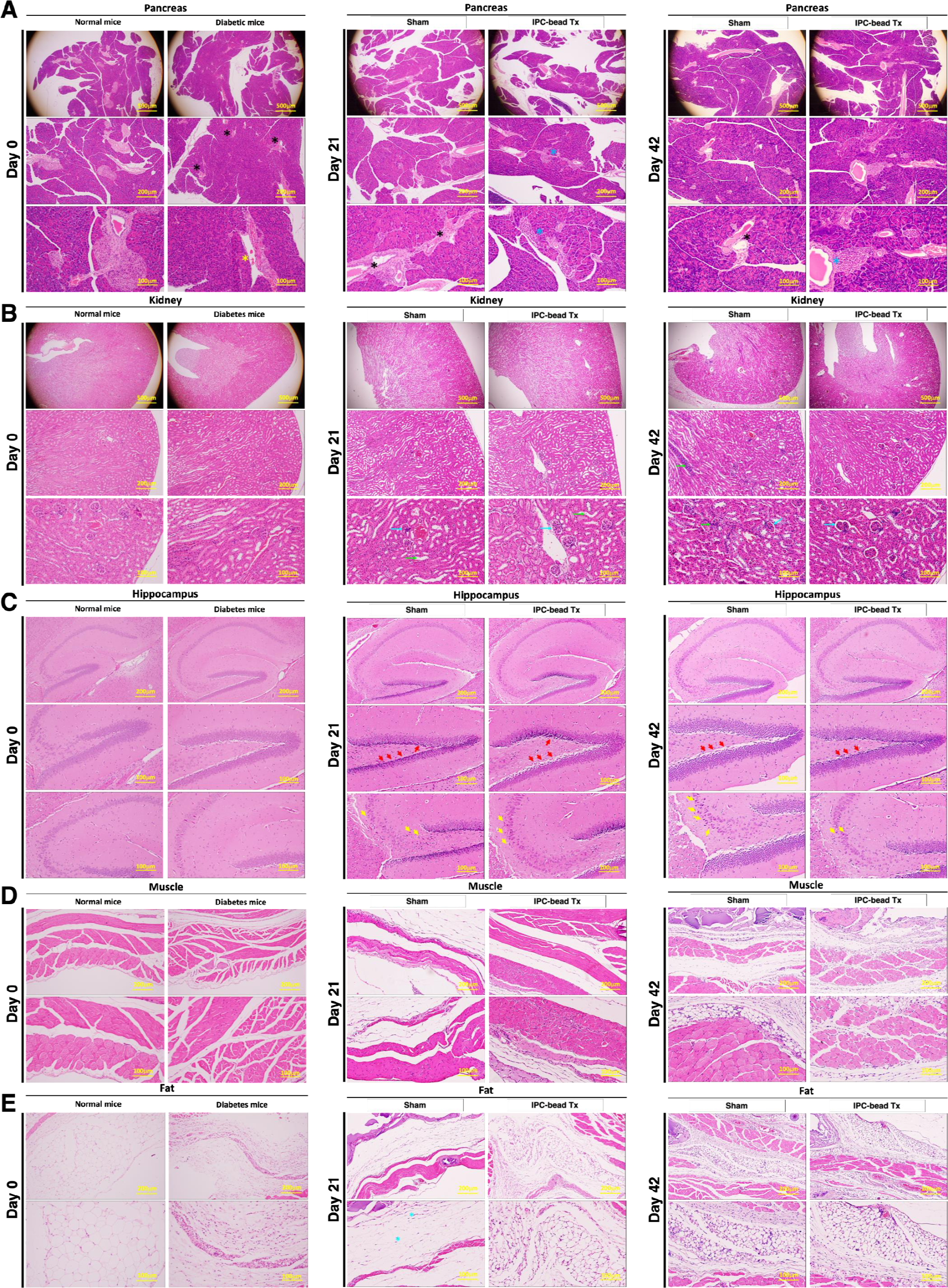

Supplement: S9 Fig — (A), (B), (C), (D), (E) Histopathological examination of insulin-dependent tissues (pancreas, kidney, hippocampus, muscle, and fat) of animals undergone subcutaneous IPC-bead transplantation in an induced type I diabetic mouse model at day 0, 21, and 42 of the study using hematoxylin & eosin (H&E) staining. Annotations: black asterisks: interlobular duct, yellow asterisks: pancreatic parenchyma, blue asterisks: regenerative islets, bright blue arrows: glomeruli and interstitial areas, bright green: tuberous, short red arrows: dentate gyrus, short yellow arrows: CA2, CA3 areas, bright blue asterisks: adipose cells. (TIF) [file pone.0318204.s009.tif]

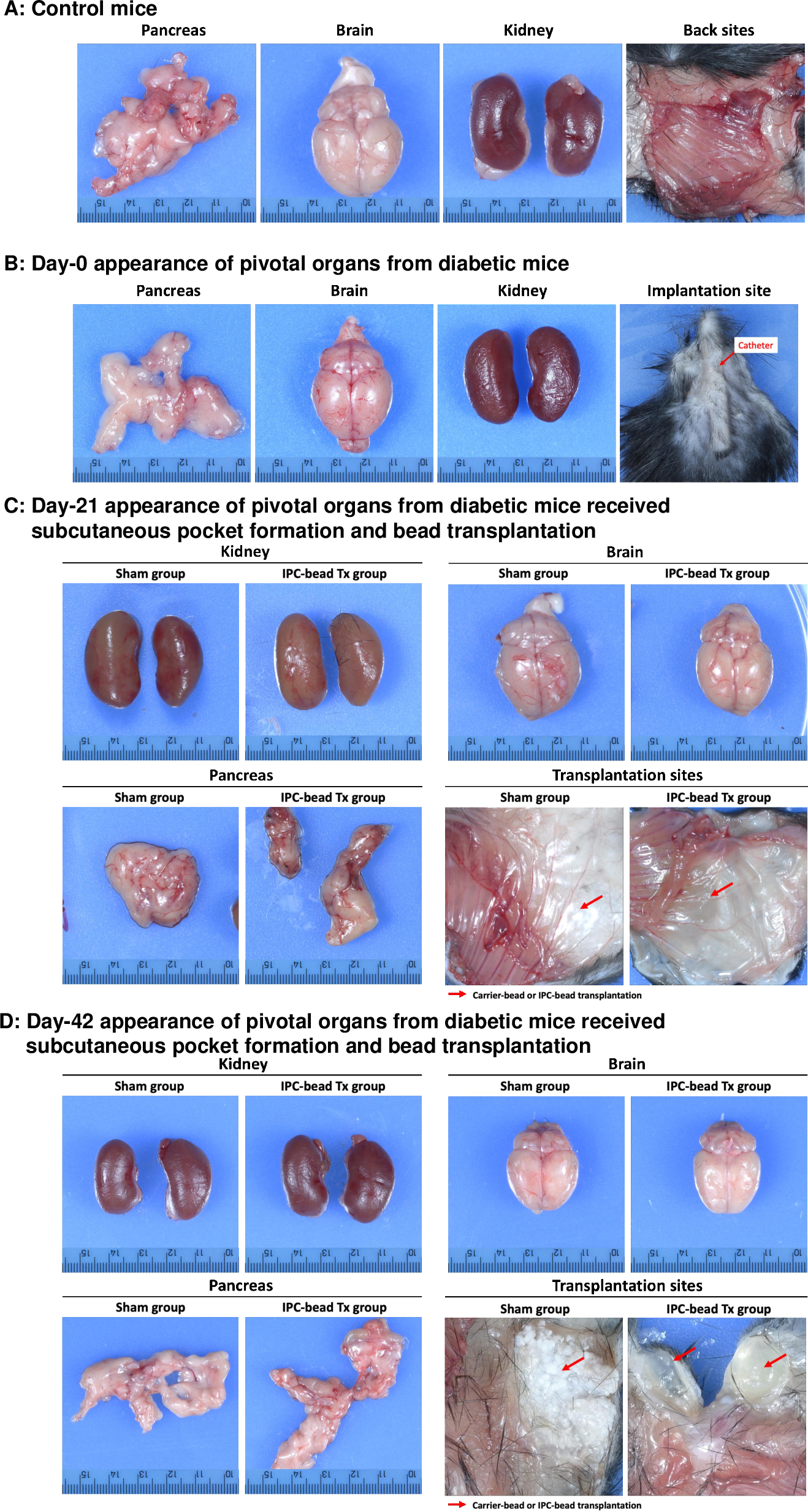

Supplement: S10 Fig — Reprinted from “Materials for supplementary figures: Establishment of subcutaneous transplantation platform for delivering induced pluripotent stem cell-derived insulin-producing cells” under a CC BY license, with permission from Chenphop Sawangmake, original copyright 2024. (TIF) [file pone.0318204.s010.tif]
