# Supplementary material for: Establishment of subcutaneous transplantation platform for delivering induced pluripotent stem cell-derived insulin-producing cells
Source: PLoS One. 2025 Jan 30;20(1):e0318204. doi: 10.1371/journal.pone.0318204 (PMC11781742; doi:10.1371/journal.pone.0318204)
Supplement: S1 Table — (PDF) [file pone.0318204.s011.pdf]

S1 Table. Primer sequences

No.	Gene	Accession # (mRNA)	Forward Primer sequence (5' - 3')	Reverse Primer sequence (5' - 3')	Primer size
1	*Rex-1*	NM_009556.3	GGAGGAAATAGGTAGAGCGCA	AGTGAGGCGATCCTGCTTTC	21
2	*Nanog*	NM_001289828.1	GGTGTCTTGCTCTTTCTGTGG	TGTCAGTGTGATGGCGAGG	21
3	*Oct-4*	NM_001252452.1	GCAGATAGGAACTTGCTGGGT	AAGCGACAGATGGTGGTCTG	21
4	*Sox-2*	NM_011443.4	TTGGGAGGGGTGCAAAAAGA	TTCTAGTCGGCATCACGGTT	20
5	*Ssea1 (Fut4)*	NM_010242.3	ACGTGTCTGTGGACGTGTTT	ACGTGCCGTGAGTTCTCAAA	20
6	*Cxcr4*	NM_001356509.1	GCGTTTGGTGCTCCGGTAAC	GAAGCAGGGTTCCTTGTTGGA	20
7	*Goosecoid (Gsc)*	NM_010351.1	TCCAGGAGACGAAGTACCCA	TCGGCGTTTTCTGACTCCTC	20
8	*FoxA2*	NM_001291065.1	ATGCACTCGGCTTCCAGTATG	TGTTCATGCCATTCATCCCCA	21
9	*Sox17*	NM_001289464.1	CCCCAAGGCTAGCTTCCGAT	TCTGGTCGTCACTGGCGTAT	20
10	*Bmp2*	NM_007553.3	GCTAGATCTGTACCGCAGGC	GAAGTTCCTCCACGGCTTCT	20
11	*Pdx1*	NM_008814.4	CCTTTCCCGAATGGAACCGA	TTCCGCTGTGTAAGCACCTC	20
12	*Sox9*	NM_011448.4	AAGAGACCCTTCGTGGAGGA	ATGTGAGTCTGTTCCGTGGC	20
13	*Hnf6 (Onecut1)*	NM_008262.3	GCAACGTGAGCGGTAGTTTC	GTCCTTGCTGGGAGTTGTGA	20
14	*Gp2*	NM_025989.4	CTGGGCAGGGAGGAAGGATAC	TCACAACCCACCATCCTTTTCA	21
15	*Ngn-3*	NM_009719.6	GCTGCTTGACACTGACCCTA	AGGTGGGGTGGAATTGGAAC	20
16	*NeuroD1*	NM_010894.3	GCCTTTACCATGCACTACCCT	GATGGCATTAAGCTGGGCAC	21
17	*Pax-4*	NM_001159925.1	CACAGCTGCCAGGGATCATCT	ATAGGCCTGGGATGAGGTGT	21
18	*Nkx-2.2*	NM_001077632.1	TCGCTACAAGATGAAACGTGC	CTTGCGGACACTATGGGCA	21
19	*Maf-b*	NM_010658.3	GGCAACTAACGCTGCAACTC	CGGAAGGGACTTGAACACCA	20
20	*Pax-6*	NM_001244198.2	GCTTTGAGAAGTGTGGGAACC	AATACGGGGCTCTGAGAACTG	21
21	*Nkx-6.1*	NM_144955.2	GCACGCTTGGCCTATTCTCT	TTCGGGTCCAGAGGTTTGTT	20
22	*Isl1*	NM_021459.4	CCCAGAGTCATCCGAGTGTG	GAGTTCCTGTCATCCCCTGG	20

23	*Mafa*	NM_194350.2	CGCACCCGACTTCTTTCTGT	CAGAGTCTGAACCGAGACCG	20
24	*Glut2*	NM_031197.2	ACCGGGATGATTGGCATGTT	GGACCTGGCCCAATCTCAAA	20
25	*Insulin1 (Ins1)*	NM_008386.4	AATGGGCCAAACAGCAAAGT	TAGGAAGTGCACCAACAGGG	20
26	*Insulin2 (Ins2)*	NM_001185083.2	GCAAGCAGGAAGGTTATTGTTTCA	CACACACCAGGTAGAGAGCC	24
27	*Glucagon*	NM_008100.4	TCTACACCTGTTCGCAGCTC	GTCCTCATGCGCTTCTGTCT	20
28	*Glp1 receptor (Glp1r)*	NM_021332.2	GGGCCAGTAGTGTGCTACAA	CTTCACACTCCGACAGGTCC	20
29	*Dll1*	NM_007865.3	AGATAACCCTGACGGAGGCT	ACCGGCACAGGTAAGAGTTG	20
30	*Jagged1*	NM_013822.5	CGGGGGTAACACCTTCAATCT	TCCACCAGCAAAGTGTAGGAC	21
31	*Jagged2*	NM_010588.2	CCTCGTCGTCATTCCCTTTCA	CAGCTCCTCATCTGGAGTGGT	21
32	*Hes1*	NM_008235.2	AGAAAGATAGCTCCCGGCAT	GTCACCTCGTTCATGCACTC	20
33	*Hes5*	NM_001370755.1	AAGGCCGACATCCTGGAGAT	GTGCAGGGTCAGGAACTGTA	20
34	*Hey1*	NM_010423.2	GTTAACTCCTCCTTGCCCGC	TCGTTGGGGACATGGAACAC	20
35	*Lef1*	NM_001276402.1	GACTTCAGGTGGTAAGAGAAGC	TGTCAGTGTTCCTTGGGGTC	22
36	*Tcf7*	XM_030245770.1	TTTCCCGGACAAACTTCCAGA	GTTATGCAGCGGGGGTTGAG	21
37	*Rpl13a*	NM_009438.5	TGAATACCAACCCCTCCCGA	CTCTCTTGGTCTTGTGGGGC	20

Rex1, reduced expression 1; ***Nanog***, Nanog homeobox; ***Oct-4***, octamer transcription factor 4; ***Sox-2,*** SRY (sex determining region Y)-box 2; ***Ssea1***, Stage-specific embryonic antigen 1; ***Cxcr4***, C-X-C chemokine receptor type 4; ***Goosecoid*** **(*Gsc*),** Homeobox protein goosecoid; ***FoxA2***, Forkhead box protein A2; ***Sox17***, SRY-box transcription factor 17; ***Bmp2***, bone morphogenetic protein 2; ***Pdx1***, pancreatic and duodenal homeobox 1; ***Sox9***, SRY-Box Transcription Factor 9; ***Hnf6***, ONECUT1 one cut homeobox 1; ***Gp2***, glycoprotein 2; ***Ngn-3***, Neurogenin-3 is a marker of embryonic-type endocrine progenitor cells; ***NeuroD1***, neurogenic differentiation 1; ***Pax-4***, Paired Box 4; ***Nkx-2.2***, Homeobox protein; ***Maf-b***, MAF bZIP transcription factor; ***Pax-6***, paired box 6; ***Nkx-6.1***, NK6 homeobox 1; **Isl1**, ISL LIM homeobox 1; ***Mafa***, MAF BZIP Transcription Factor ; ***Glut2***, Slc2a2 solute carrier family 2; ***Insulin1*** (Ins1), ***Insulin2*** (Ins2), ***Glucagon***, ***Glp1 receptor*** (*Glp1r*), glucagon-like peptide 1 receptor; ***Dll1***, delta like canonical Notch ligand 1; ***Jagged1***, jagged canonical Notch ligand 1; ***Jagged2***, jagged canonical Notch ligand 2; ***Hes1***, hairy and enhancer of split-1 or hes family bHLH transcription factor 1; ***Hes5***, hairy and enhancer of split-5 or hes family bHLH transcription factor 5; ***Hey1***, hes related family bHLH transcription factor with YRPW motif 1; ***Lef1***, lymphoid enhancer binding factor 1; ***Tcf7***, transcription factor 7, T cell specific; ***Rpl13a***, ribosomal protein L13A.
