# Supplementary material for: Establishment of subcutaneous transplantation platform for delivering induced pluripotent stem cell-derived insulin-producing cells
Source: PLoS One. 2025 Jan 30;20(1):e0318204. doi: 10.1371/journal.pone.0318204 (PMC11781742; doi:10.1371/journal.pone.0318204)
Supplement: S2 Table — (PDF) [file pone.0318204.s012.pdf]

S2 Table. Complete blood count parameters of animals undergone subcutaneous IPC transplantation platform establishment.

Parameter	Day 0		Day 21		Day 42		Ranges	Units
	CTRL (n=3)	Carrier-bead (n=3)	CTRL (n=3)	Carrier-bead (n=3)	CTRL (n=1)	Carrier-bead (n=1)		
**HB**	14.10 ± 0.643	12.70 ± 1.686	13.70 ± 0.987	14.40 ± 0.681	14.90	13.70	6.10 - 21.70	g/dL
**HCT**	50.20 ± 2.685	45.00 ± 6.322	49.40 ± 4.537	52.70 ± 3.721	53.40	48.90	16.70 - 69.80	%
**WBC animal count**	7.50 ± 1.957	6.10 ± 0.185	6.10 ± 0.606	7.10 ± 1.735	6.46	6.22	1.06 - 56.08	10^3*cell/µL
**RBC animal count**	9.60 ± 0.604	8.70 ± 1.089	9.30 ± 0.707	10.10 ± 0.627	10.05	9.92	3.57 - 15.2	10^6*cell/µL
**PMN**	3.00 ± 1.000	2.70 ± 0.577	3.00 ± 0.000	3.70 ± 0.577	2.00	10.00	4.27 - 18.48	%
**LYMPHOCYTE**	92.00 ± 1.000	91.30 ± 0.577	91.70 ± 0.577	88.30 ± 2.082	77.00	78.00	71.77 - 89.94	%
**MONOCYTE**	0.30 ± 0.577	0.70 ± 0.577	0.70 ± 0.577	1.00 ± 0.000	1.00	1.00	0.00 - 5.08	%
**EOSINOPHIL**	0.30 ± 0.577	0.00	0.00	0.70 ± 1.155	0.00	1.00	0.00 - 2.03	%
**BASOPHIL**	4.30 ± 0.577	5.30 ± 0.577	4.70 ± 0.577	6.30 ± 0.577	20.00	10.00	0.00 -2 .33	%
**RBC MORPHOLOGY**	**Normochromic/Normocytic**							
**MCV**	52.10 ± 0.896	51.60 ± 1.058	52.90 ± 1.193	52.20 ± 1.179	53.10	49.30	39.00 - 90.8	fL
**MCH**	14.60 ± 0.252	14.60 ± 0.231	14.70 ± 0.173	14.30 ± 0.300	14.80	13.80	12.6.0 – 31.00	pg
**MCHC**	28.00 ± 0.252	28.20 ± 0.200	27.80 ± 0.611	27.40 ± 0.651	27.90	28.00	27.00 - 37.60	g/dL
**BLOOD PARASITE**	**Not found**							
**PLATELET COUNT**	504.00 ± 45.738	238.30 ± 257.115	258.33 ± 22.85	315.00 ± 96.161	974.00	978.00	59.00 – 2633.00	10^3*cell/µL

HB: Hemoglobin; HCT: Hematocrit; WBC: White Blood Cells; PMNs: Polymorphonuclear leukocytes; RBC: Red Blood Cells; MCV: Mean Corpuscular Volume; MCH: Mean Corpuscular Hemoglobin; MCHC: Mean Corpuscular Hemoglobin Concentration.
