# Supplementary material for: Establishment of subcutaneous transplantation platform for delivering induced pluripotent stem cell-derived insulin-producing cells
Source: PLoS One. 2025 Jan 30;20(1):e0318204. doi: 10.1371/journal.pone.0318204 (PMC11781742; doi:10.1371/journal.pone.0318204)
Supplement: S3 Table — (PDF) [file pone.0318204.s013.pdf]

S3 Table. Blood chemistry parameters of animals undergone subcutaneous IPC transplantation platform establishment.

Parameter	Day 0		Day 21		Day 42		Ranges	Unit
	CTRL (n=3)	Carrier-bead (n=3)	CTRL (n=3)	Carrier-bead (n=3)	CTRL (n=3)	Carrier-bead (n=3)		
**ALB**	4.80 ± 0.173	4.60 ± 0.100	4.90 ± 0.153	4.30 ± 0.354	4.57 ± 0.321	4.367 ± 0.321	2.6-5.4	g/dL
**ALP**	62.30 ± 7.506	47.30 ± 11.930	33.70 ± 20.744	32.00 ± 12.728	42.00 ± 24.576	19.00 ± 5.568	16-200	U/L
**ALT**	40.00 ± 4.583	107.30 ± 60.715	58.70 ± 22.301	48.50 ± 6.364	253.70 ± 191.398	136.00 ± 104.312	22-133	U/L
**AMY**	994.00 ± 70.633	1442.70 ± 820.488	991.70 ± 52.205	900.00 ± 2.828	1587.00 ± 942.813	975.33 ± 44.015	608-2000	U/L
**TBIL**	0.30 ± 0.000	0.30 ± 0.058	0.20 ± 0.058	0.30 ± 0.071	0.28 ± 0.058	0.23 ± 0.058	0.1-0.9	mg/dL
**BUN**	19.70 ± 1.155	19.30 ± 1.528	18.00 ± 1.000	20.00 ± 0.000	26.00 ± 5.292	18.33 ± 3.215	2.0-71	mg/dL
**CA**	11.60 ± 0.586	11.50 ± 0.520	11.50 ± 0.451	10.50 ± 1.273	10.60 ± 2.343	10.07 ± 3.272	6.8-11.9	mg/dL
**PHOS**	18.20 ± 3.053	16.40 ± 2.411	9.00 ± 1.580	10.10 ± 0.354	14.13 ± 2.223	14.53 ± 2.055	6.0-11.3	mg/dL
**CRE**	0.30 ± 0.058	0.20 ± 0.058	0.20 ± 0.115	0.40 ± 0.071	0.43 ± 0.058	0.10 ± 0.100	0.1-1.8	mg/dL
**GLU**	142.70 ± 17.559	181.70 ± 26.388	278.00 ± 60.893	211.50 ± 38.891	288.67 ± 54.930	339.67 ± 44.072	114-279	mg/dL
**NA^+**	161.30 ± 4.163	161.30 ± 2.309	151.00 ± 2.646	144.50 ± 10.607	154.67 ± 3.055	154.00 ± 1.000	153-175	mmol/L
**K^+**	10.20 ± 1.234	10.50 ± 0.404	10.80 ± 0.451	10.50 ± 0.778	9.87 ± 1.305	9.70 ± 0.265	6.5-9.7	mmol/L
**Total Protein**	5.90 ± 0.173	5.70 ± 0.173	5.90 ± 0.252	5.30 ± 0.849	6.00 ± 0.265	5.80 ± 0.173	4.6-7.3	g/dL
**β-GLOB**	1.10 ± 0.000	1.10 ± 0.321	1.10 ± 0.115	1.10 ± 0.424	1.43 ± 0.306	1.43 ± 0.416	0.67-1.21	g/dL

ALB: Albumin; ALP: Alkaline Phosphatase; ALT: Alanine aminotransferase; AMY: Amylase; TBIL: Total bilirubin; BUN: Blood urea nitrogen; CA: Calcium; PHOS: Phosphorus; CRE: Creatinine; GLU: Glucose; NA+: Sodium, K+: Potassium; TP: Total protein; β-GLOB: beta Globulin.
