# Supplementary material for: Establishment of subcutaneous transplantation platform for delivering induced pluripotent stem cell-derived insulin-producing cells
Source: PLoS One. 2025 Jan 30;20(1):e0318204. doi: 10.1371/journal.pone.0318204 (PMC11781742; doi:10.1371/journal.pone.0318204)
Supplement: S4 Table — (PDF) [file pone.0318204.s014.pdf]

S4 Table. Complete blood count parameters of a 7-day validation of subcutaneous pocket formation using 10% Pluronic acid in normal mice.

Parameter	CTRL	10% Pluronic	Ranges	Units
HB	14.1	10.5	6.1-21.7	g/dL
HCT	55.1	43.8	16.7-69.8	%
WBC	7.63	6.15	1.06-56.08	10^3*cell/µL
PMN	4	5	4.27-18.48	%
LYMPHOCYTE	87	80	71.77-89.94	%
MONOCYTE	1	2	0-5.08	%
EOSINOPHIL	1	3	0-2.03	%
BASOPHIL	7	10	0-2.33	%
RBC MORPHOLOGY	Normochromic/Normocytic			
MCV	57.1	57.2	39-90.8	fL
MCH	14.6	13.7	12.6-31	pg
MCHC	25.6	24	27-37.6	g/dL
BLOOD PARASITE	Not found			
PLATELET COUNT	156	277	59-2633	cell/µL

HB: Hemoglobin; HCT: Hematocrit; WBC: White Blood Cells; PMNs: Polymorphonuclear leukocytes; RBC: Red Blood Cells; MCV: Mean Corpuscular Volume; MCH: Mean Corpuscular Hemoglobin; MCHC: Mean Corpuscular Hemoglobin Concentration.
