# Supplementary material for: Establishment of subcutaneous transplantation platform for delivering induced pluripotent stem cell-derived insulin-producing cells
Source: PLoS One. 2025 Jan 30;20(1):e0318204. doi: 10.1371/journal.pone.0318204 (PMC11781742; doi:10.1371/journal.pone.0318204)
Supplement: S5 Table — (PDF) [file pone.0318204.s015.pdf]

S5 Table. Blood chemistry parameters of a 7-day validation of subcutaneous pocket formation using 10% Pluronic acid in normal mice.

Parameter	CTRL	10% Pluronic	Range	Unit
ALB	2.9 ± 0.14	2.8 ± 0.14	2.6-5.4	g/dL
ALT	25.5 ± 0.71	21.5 ± 2.12	22-133	U/L
ALP	94.5 ± 0.71	87.5 ± 4.95	16-200	mg/dL
TBIL	0.4 ± 0.14	0.4 ± 0.14	0.1-0.9	mg/dL
Direct BIL	0.1 ± 0.0	0.15 ± 0.07	0.1-0.2	mg/dL
BUN	22 ± 1.41	20.5 ± 0.71	2.0-71	mg/dL
CRE	0.3 ± 0.0	0.25 ± 0.07	0.1-1.8	mg/dL
Total Protein	5.3 ± 0.14	5.2 ± 0.14	4.6-7.3	g/dL

ALB: Albumin; ALT: Alanine aminotransferase; ALP: Alkaline Phosphatase; TBIL: Total bilirubin; Direct BIL: Bilirubin; BUN: Blood urea nitrogen; CRE: Creatinine
