# Supplementary material for: Establishment of subcutaneous transplantation platform for delivering induced pluripotent stem cell-derived insulin-producing cells
Source: PLoS One. 2025 Jan 30;20(1):e0318204. doi: 10.1371/journal.pone.0318204 (PMC11781742; doi:10.1371/journal.pone.0318204)
Supplement: S6 Table — (PDF) [file pone.0318204.s016.pdf]

S6 Table. Complete blood count parameters of animals undergone subcutaneous IPC-bead transplantation in an induced type I diabetic mouse model.

Parameter	Day -21	Day 0	Day 21		Day 42		Ranges	Unit
	Normal mice (n=5)	Diabetic mice (n=5)	Sham (n=3)	IPC-bead Tx (n=3)	Sham (n=3)	IPC-bead Tx (n=3)		
HB	14.40 ± 1.564	13.63 ± 1.363	13.90 ± 0.755	15.67 ± 0.231	15.73 ± 0.252	16.07 ± 0.666	2.6-5.4	g/dL
HCT	46.40 ± 6.865	47.98 ± 5.279	41.70 ± 2.265	51.30 ± 5.910	52.33 ± 6.568	51.10 ± 1.308	16-200	U/L
WBC animal	9.30 ± 3.436	6.35 ± 0.861	6.00 ± 0.000	6.16 ± 0.283	7.66 ± 2.244	6.77 ± 0.893	22-133	U/L
RBC animal	9.60 ± 0.926	9.00 ± 0.919	10.08 ± 0.546	10.98 ± 0.101	11.27 ± 0.087	11.39 ± 0.618	608-1200	U/L
PMN	5.40 ± 1.517	29.50 ± 25.130	10.00 ± 5.000	8.67 ± 3.512	41.67 ± 19.655	26.33 ± 2.887	0.1-0.9	mg/dL
LYMPHOCYTE	90.40 ± 1.517	64.33 ± 23.872	57.33 ± 14.742	53.00 ± 15.875	46.00 ± 21.166	69.33 ± 7.024	2.0-71	mg/dL
MONOCYTE	0.40 ± 0.548	5.33 ± 8.710	7.67 ± 6.429	5.67 ± 1.528	1.67 ± 1.155	3.00 ± 3.464	6.8-11.9	mg/dL
EOSINOPHIL	0.00	0.00	0.00	0.67 ± 1.155	0.00 ± 0.000	0.67 ± 1.155	6.0-11.3	mg/dL
BASOPHIL	3.80 ± 2.490	0.83 ± 1.169	25.00 ± 10.149	32.00 ± 12.166	10.67 ± 2.082	0.67 ± 0.577	0.1-1.8	mg/dL
RBC MORPHOLOGY	Normochromic/Normocytic							
MCV	48.20 ± 3.865	53.32 ± 0.760	38.03 ± 6.012	46.73 ± 5.341	44.57 ± 2.542	45.00 ± 3.724	153-175	mmol/L
MCH	15.00 ± 0.381	15.17 ± 0.463	13.77 ± 0.115	14.27 ± 0.252	13.97 ± 0.231	14.10 ± 0.173	6.5-9.7	mmol/L
MCHC	31.30 ± 1.741	28.47 ± 1.271	25.13 ± 0.208	28.87 ± 3.272	32.63 ± 0.058	31.50 ± 2.078	4.6-7.3	g/dL
BLOOD PARASITE	Not found							
PLATELET COUNT	464.00 ± 362.724*	728.67 ± 265.340*	950.00 ± 77.660*	798.00 ± 240.576*	936.53 ± 54.669*	665.67 ± 428.724*	59.00 – 2633.00	10^3*cell/µL

HB: Hemoglobin; HCT: Hematocrit; WBC: White Blood Cells; PMNs: Polymorphonuclear leukocytes; RBC: Red Blood Cells; MCV: Mean Corpuscular Volume; MCH: Mean Corpuscular Hemoglobin; MCHC: Mean Corpuscular Hemoglobin Concentration.
*Annotation: *: significant difference*
