# Supplementary material for: Establishment of subcutaneous transplantation platform for delivering induced pluripotent stem cell-derived insulin-producing cells
Source: PLoS One. 2025 Jan 30;20(1):e0318204. doi: 10.1371/journal.pone.0318204 (PMC11781742; doi:10.1371/journal.pone.0318204)
Supplement: S7 Table — (PDF) [file pone.0318204.s017.pdf]

S7 Table. Blood chemistry parameters of animals undergone subcutaneous IPC-bead transplantation in an induced type I diabetic mouse model.

Parameter	Day -21	Day 0	Day 21		Day 42		Ranges	Unit
	Normal mice (n=5)	Diabetic mice (n=5)	Sham (n=3)	IPC-bead Tx (n=3)	Sham (n=3)	IPC-bead Tx (n=3)		
ALB	4.80 ± 0.342	3.22 ± 0.259	3.17 ± 0.586	3.63 ± 0.153	4.03 ± 0.208	3.87 ± 0.153	2.6-5.4	g/dL
ALP	54.00 ± 12.349	126.60 ± 50.693	127.33 ± 8.386	83.33 ± 72.176	136.33 ± 17.786	133.67 ± 13.051	16-200	U/L
ALT	98.20 ± 32.337*	141.00 ± 28.700*	790.33 ± 670.709*	139.00 ± 66.189*	149.00 ± 70.739*	106.00 ± 8.000*	22-133	U/L
AMY	1010.20 ± 253.820*	820.00 ± 193.808*	626.33 ± 214.311*	896.33 ± 308.234	737.00 ± 61.147*	1131.33 ± 488.848	608-1200	U/L
TBIL	0.20 ± 0.055	0.36 ± 0.251	0.33 ± 0.06	0.33 ± 0.058	0.30 ± 0.000	0.30 ± 0.000	0.1-0.9	mg/dL
BUN	19.60 ± 1.817	52.20 ± 17.370	68.33 ± 26.312	42.00 ± 13.856	27.67 ± 8.021	37.67 ± 8.505	2.0-71	mg/dL
CA	12.00 ± 0.400	10.98 ± 0.589	10.47 ± 0.416	7.90 ± 4.678	11.67 ± 0.208	11.00 ± 0.173	6.8-11.9	mg/dL
PHOS	11.80 ± 1.818	16.60 ± 9.673	12.40 ± 0.400	11.47 ± 1.815	13.80 ± 2.193	11.50 ± 1.473	6.0-11.3	mg/dL
CRE	0.00 ± 0.045	0.16 ± 0.251	0.17 ± 0.153	0.23 ± 0.208	0.07 ± 0.058	0.27 ± 0.462	0.1-1.8	mg/dL
GLU	261.60 ± 50.058*	929.00 ± 61.278*	973.67 ± 45.611*	952.00 ± 78.000*	631.33 ± 31.390*	786.00 ± 103.523*	114-279	mg/dL
NA+	157.40 ± 3.050	154.40 ± 3.435	151.00 ± 4.000	153.00 ± 4.359	151.00 ± 1.000	146.67 ± 1.528	153-175	mmol/L
K+	9.00 ± 0.923	9.86 ± 2.519	9.97 ± 0.651	8.40 ± 4.851	10.90 ± 0.100	10.23 ± 1.159	6.5-9.7	mmol/L
TP	6.00 ± 0.295	4.28 ± 0.466	4.47 ± 0.416	4.53 ± 0.208	5.23 ± 0.306	5.13 ± 0.115	4.6-7.3	g/dL
β-GLOB	1.20 ± 0.195	1.10 ± 0.316	1.30 ± 0.755	0.90 ± 0.100	1.23 ± 0.115	1.27 ± 0.058	0.67-1.21	g/dL

ALB: Albumin; ALP: Alkaline Phosphatase; ALT: Alanine aminotransferase; AMY: Amylase; TBIL: Total bilirubin; BUN: Blood urea nitrogen; CA: Calcium; PHOS: Phosphorus; CRE: Creatinine; GLU: Glucose; NA+: Sodium, K+: Potassium; TP: Total protein; β-GLOB: beta Globulin.

*Annotation: *: significant difference*
